# Fifteen Marseilleviruses Newly Isolated From Three Water Samples in Japan Reveal Local Diversity of *Marseilleviridae*

**DOI:** 10.3389/fmicb.2019.01152

**Published:** 2019-05-24

**Authors:** Keita Aoki, Reika Hagiwara, Motohiro Akashi, Kenta Sasaki, Kazuyoshi Murata, Hiroyuki Ogata, Masaharu Takemura

**Affiliations:** ^1^Laboratory of Biology, Graduate School of Mathematics and Science Education, Tokyo University of Science, Tokyo, Japan; ^2^Laboratory of Biology, Department of Liberal Arts, Faculty of Science, Tokyo University of Science, Tokyo, Japan; ^3^National Institute for Physiological Sciences, Okazaki, Japan; ^4^Institute for Chemical Research, Kyoto University, Gokasho, Japan

**Keywords:** giant viruses, family *Marseilleviridae*, phylogenetic analysis, major capsid protein, nucleo-cytoplasmic large DNA virus, isolation

## Abstract

The family *Marseilleviridae*, defined as a group of icosahedral double-stranded DNA viruses with particle size of approximately 250 nm and genome size of 350–380 kbp, belongs to the nucleo-cytoplasmic family of large DNA viruses. The family *Marseilleviridae* is currently classified into lineages A–E. In this study, we isolated 12 or 15 new members of the family *Marseilleviridae* from three sampling locations in Japan. Molecular phylogenetic analysis of the MCP genes showed that the new viruses could be further classified into three groups, hokutoviruses, kashiwazakiviruses, and kyotoviruses. Hokutoviruses were closely related to lineage B, kyotoviruses were related to lineage A, and kashiwazakiviruses were also classified into lineage B but a new putative subgroup of lineage B, revealing the diversity of this lineage. Interestingly, more than two viruses with slightly different MCP genes were isolated from a single water sample from a single location, i.e., two hokutoviruses and one kashiwazakivirus were isolated from a small reservoir, five kashiwazakiviruses from the mouth of a river, and five kyotoviruses from fresh water of a river, suggesting that several milliliters of water samples contain several types of giant viruses. Amoeba cells infected with hokutoviruses or kashiwazakiviruses exhibited a “bunch” formation consisting of normal and infected cells similarly to a tupanvirus, whereas cells infected with kyotoviruses or tokyovirus did not. These results suggest the previously unrecognized local diversity of the family *Marseilleviridae* in aquatic environments.

## Introduction

During the past two decades, nucleo-cytoplasmic large DNA viruses (NCLDVs) have been found to form diverse groups and families of viruses. Among them, the number of so-called “giant viruses” identified has drastically increased since the discovery of the first giant virus, *Acanthamoeba polyphaga mimivirus*, in 2003 (La Scola et al., 2003). Several groups of giant viruses, such as the family *Mimiviridae*, family *Marseilleviridae*, pandoraviruses, pithoviruses, mollivirus, and medusavirus, have been isolated from various environments worldwide (La Scola et al., 2003; Boyer et al., 2009; Legendre et al., 2013, 2014, 2015; Philippe et al., 2013; Yoshikawa et al., 2019). Among them, the family *Mimiviridae* and family *Marseilleviridae* have been recognized by the International Committee on Taxonomy of Viruses. The family *Marseilleviridae* is comprised of icosahedral double-stranded DNA viruses with particle sizes of approximately 250 nm and genome sizes of 350–380 kbp (Boyer et al., 2009; Colson et al., 2013). The founder of the family *Marseilleviridae, Marseillevirus marseillevirus*, was isolated from a cooling tower in Paris (Boyer et al., 2009). Since then, many members of the family *Marseilleviridae* have been discovered not only in a variety of aquatic environments, but also in the human intestines and insect bodies (Lagier et al., 2012; Boughalmi et al., 2013). The family *Marselleviridae* is currently classified into several lineages (Chatterjee and Kondabagil, 2017; Fabre et al., 2017): *Marseillevirus marseillevirus* (Boyer et al., 2009), melbournvirus (Doutre et al., 2014), Cannes 8 virus (Aherfi et al., 2013), senegalvirus (Lagier et al., 2012), tokyovirus (Takemura, 2016), and *Marseillevirus shanghai* are classified in lineage A; lausannevirus (Thomas et al., 2011), Port-miou virus (Doutre et al., 2015), noumeavirus (Fabre et al., 2017), and kurlavirus (Chatterjee and Kondabagil, 2017) are classified in lineage B; tunisvirus (Aherfi et al., 2014) and insectomime virus (Boughalmi et al., 2013) are classified in lineage C; Brazilian marseillevirus (Dornas et al., 2016) are classified in lineage D; and golden marseillevirus (Dos Santos et al., 2016) are classified in lineage E.

In addition to conventional methods for isolating viruses, various giant virus genomes have recently been detected in oceans, rivers, and soil environments by metagenomic analysis (Law et al., 2013; Martínez et al., 2014). Through metagenomics, it has been suggested that there are multiple types of giant viruses even in a single environmental sample (Li et al., 2018; Schulz et al., 2018). However, cases of isolated giant viruses, such as the family *Marseilleviridae*, reported so far have shown a trend of “one virus per one sample.” Thus, the existence of multiple types of giant virus in a single water sample from a single location has only been clarified using metagenomic analysis rather than “classical” isolation methods (Zhang et al., 2015; Verneau et al., 2016; Roux et al., 2017; Schultz et al., 2017; Andreani et al., 2018; Schulz et al., 2018). Using metagenomics analysis in our previous study, we identified many giant viruses; however, we could not analyze their phenotypes. Therefore, we isolated multiple types of giant viruses from a single environmental sample using classical isolation methods and focused on their major capsid protein (MCP) gene.

## Materials and Methods

### Virus Isolation

*Acanthamoeba castellanii* (Douglas) Neff (ATCC 30010^TM^) cells were cultured in PYG (proteose peptone – yeast extract – glucose) medium as previously described (Takemura, 2016; Yoshikawa et al., 2019). Water samples were collected from the small reservoir near Hokuto Town in Kashiwazaki City, Niigata; from the mouth of the Ukawa River in Kashiwazaki City; and from the Tatakaigawa River in Uji City, Kyoto. For two water samples from Kashiwazaki City, 4.5 mL of 2× PYG medium, 4.5 mL of the water sample, 360 μL of a mixed solution of antibiotics, and 50 μL of the amoeba culture solution (approximately 1.5 × 10^5^ cells) were added to a 15-mL centrifuge tube and mixed with gentle rotation. Next, 100 μL of this sample was seeded into a 96-well microplate and incubated at 26°C. After 2–3 days, the cytopathic effect (CPE) was detected from three wells from the small reservoir near Hokuto Town and five wells of inoculated samples from the Ukawa River. The supernatants of these wells were then inoculated into fresh amoeba cells to propagate putative viruses in each sample.

For the water sample from the Tatakaigawa River in Kyoto, mud was removed by filtration through a filter paper with a pore size of 20 μm (No. 43; Whatman International, Maidstone, United Kingdom), and then the sample was further filtered using a filter paper with a pore size of 1.2 μm (Acrodic^®^ 32 mm Syringe Filter, Pall Co., Cornwall, United Kingdom). Eighteen milliliters of PYG medium were mixed with 9.5 mL of the filtered sample and 0.5 mL of the amoeba suspension (approximately 1.5 × 10^6^ cells). The mixture was incubated at room temperature for 1 h with gentle rotation. This mixture was divided and cultured in 164 wells on two 96-well microplates at 26°C. The supernatants from the seven wells showing CPE were then inoculated into fresh amoeba cells (in a 12-well culture plate) to propagate the putative viruses in each supernatant.

### Electron Microscopy

Harvested amoeba cells showing CPE were washed twice with phosphate-buffered saline, fixed in 2% glutaraldehyde solution, and stained with 2% osmium tetroxide as described previously (Takemura, 2016; Yoshikawa et al., 2019). Osmium-stained cells were dehydrated in increasing the ethanol concentrations and embedded in Epon-812 (TAAB Laboratory Equipment, Berks, United Kingdom). For the samples of hokutovirus and kashiwazakivirus, plastic-embedded amoeba cells were sectioned at 70 nm thickness using an ultramicrotome (EM-UC7; Leica Microsystems, Wetzlar, Germany), and mounted on a formvar-coated slot mesh. The meshes were stained with 2% uranyl acetate and 1% lead citrate for 5 min each. Observations were performed using a transmission electron microscope (TEM, JEM1010, JEOL, Ltd., Tokyo, Japan) operated at 80 kV. For kyotovirus, sectioning, mounting, staining, and observations were carried out by the Hanaichi Ultrastructure Research Institute (Okazaki, Aichi, Japan).

### Titration of Viruses and Observation of “Bunch” Formation of Amoebas

*Acanthamoeba castellanii* cells (approximately 10,000 cells) were added to 96-well microplates with 90 μL of PYG in each well. Ten serial dilutions were performed for each virus solution in 1.5-mL microtubes. A total of 10 μL of each diluted virus solution was added to each well. Virus titers were calculated using a TCID_50_ calculator v.2.1 (©Marco Binder, Department of Infectious Diseases, Molecular Virology, Heidelberg University). The number of amoeba cells in each well was counted using a Counting Chamber (Erma, Tokyo, Japan). The multiplicity of infection (MOI) was calculated from these two values. Again, amoeba cells were seeded into a 96-well microplate with 90 μL of PYG in each well, and 10 μL of virus solutions of various titers were added. *Acanthamoeba castellanii* cells in each well were observed at days 1, 2, and 3 after infection using a phase-contrast microscope, Eclipse TS100 (Nikon Corporation, Tokyo, Japan).

### Design of Primers for MCP Gene of the Family *Marseilleviridae*

To amplify the MCP genes of the family *Marseilleviridae*, we designed primers to detect MCP genes of several *Marseilleviridae* lineages except for golden marseillevirus. The forward primer F1 (25 bases) was designed on the 5′ end of the MCP gene including 15 bases of the 5′-untranslated region (UTR); 5′-STMYBDTKGAGAGTAATGACTTCTG-3′. One reverse primer R5 (20 bases) was designed for the 3′-UTR of the MCP gene; 5′-GGWTGCAGGRRWTGYTCMTAC-3′. Another reverse primer R6 (20 bases) was designed for the 3′-UTR of the MCP gene; 5′-CRAAKAGATGAGTGACTGRA-3′. Abbreviations of mixed bases are as follows: S = C+G, M = A+C, Y = C+T, B = C+G+T, D = A+G+T, K = G+T, and R = A+G.

### Cloning of Hokutoviruses, Kashiwazakiviruses, and Kyotoviruses

Amoeba cells were seeded into a 96-well culture plate with 90 μL of PYG in each well. Ten dilution series of virus suspension were prepared in 1.5-mL microtubes for each hokutovirus, kashiwazakivirus, and kyotovirus. A total of 10 μL diluted virus solution was added to each well. After several days, the CPE was detected in each well, and the supernatants of the most diluted well (in which CPE was detected) were collected and then inoculated into fresh amoeba cells to propagate the cloned viruses.

### Sequence Analysis of MCP Genes

After virus cloning, the genomic DNA of each hokutovirus and kashiwazakivirus was extracted from viral particles in the supernatant of virus-infected *A. castellanii* cultures according to the manufacturer’s protocol (NucleoSpin^®^ Tissue XS, Macherey-Nagel GmbH and Co. KG, Düren, Germany). Full-length MCP genes including part of the intergenic regions flanking MCP were amplified by PCR using extracted genomic DNA as a template, F1 as a forward primer, and R5 as a reverse primer. For each kyotovirus, full-length MCP genes, including part of the 5′-UTR and 3′-UTR, were amplified by PCR using virus solution as a template, F1 as a forward primer, and R6 as a reverse primer. Amplified full-length MCP genes of these viruses were sequenced using 4 primers Seq-F1, Seq-F2, Seq-R1, and Seq-R2 (Supplementary Table 1) and primers F1, R5 (hokutoviruses and kashiwazakiviruses) or R6 (kyotoviruses). Capillary sequences were performed by Fasmac Co., Ltd. (Atsugi, Japan). Full-length sequences of each MCP are shown in Supplementary Data 1.

### Phylogenetic Analysis

Nucleotide sequences of MCP genes from members of the family *Marseilleviridae, Marseillevirus marseillevirus*, lausannevirus, melbournevirus, tunisvirus, insectomime virus, Cannes 8 virus, Port-Miou virus, tokyovirus, *Marseillevirus shanghai*, Brazilian marseillevirus, golden marseillevirus, noumeavirus, and kurlavirus were obtained from the NCBI nucleotide sequence database^1^. These sequences and newly identified MCP sequences of all hokutoviruses, kashiwazakiviruses, and kyotoviruses were aligned using the ClustalW program implemented in the MEGA X software (ver.10.0.3) with default parameters (Kumar et al., 2018). To reconstruct the maximum-likelihood tree, we estimated the branch support with 1,000 bootstrap replications and used the TN93 model as a substitution model with a gamma distribution with invariant sites (G + I) as described previously (Takemura et al., 2015). We also calculated the pairwise sequence identity using the MCP gene sequence alignment. Identical sites of every virus-MCP gene were enumerated and the percentage of the total length was calculated. Sites with gaps were removed before the calculation, and 1,425 sites were used.

## Results

### Isolation and Morphology of New Viruses

**FIGURE 1:**
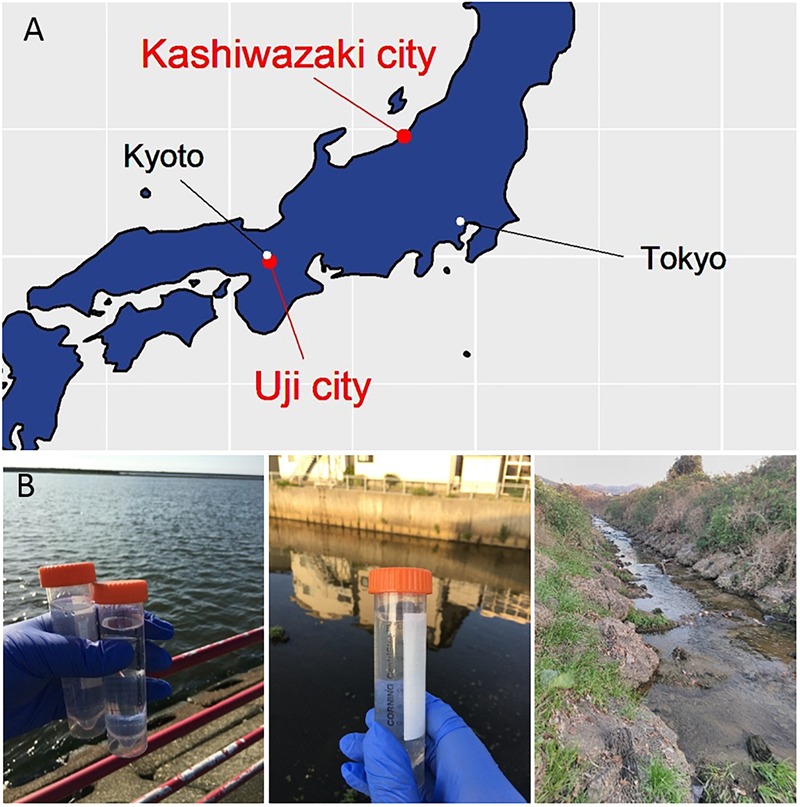
Three sampling locations. **(A)** Locations of sampling sites in Japanese island. The Ukawa River and Hokuto Town locate in Kashiwazaki City, and the Tatakaigawa locates in Uji City. **(B)** Photographs of the mouth of the Ukawa River (left panel), a small reservoir near Hokuto Town (middle panel) in Kashiwazaki City, and of the Tatakaigawa River in Uji City (right panel).

**FIGURE 2:**
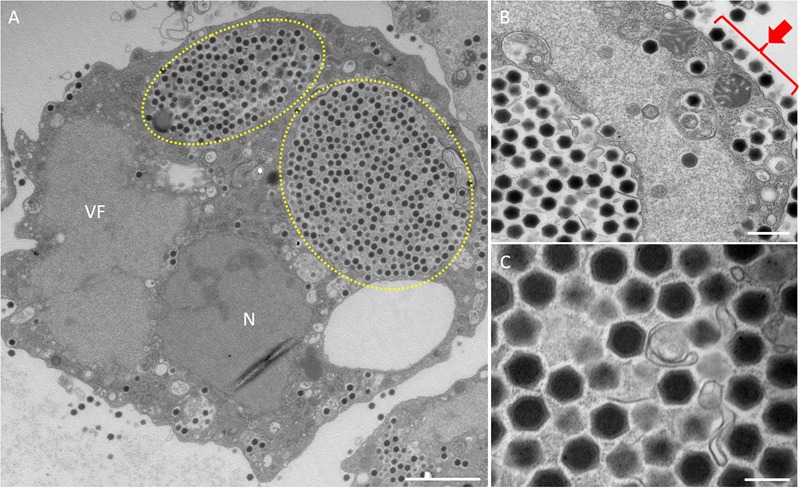
TEM images of ultrathin sections of amoeba cells infected with the new viruses isolated from a small reservoir near Hokuto Town in Kashiwazaki City (named hokutovirus). **(A)** TEM image of a typical virion factory (VF) in the cytoplasmic region of the amoeba cells. N indicates an amoeba cell nucleus. Yellow circles indicate membrane bags. Scale bar: 2 μm. **(B)** Enlarged image of virus particles in the amoeba cytoplasm. Red arrow indicates viral particles surrounding cell membrane. Scale bar: 500 nm. **(C)** Enlarged image of viral particles in the amoeba cytoplasm. Scale bar: 200 nm.

**FIGURE 3:**
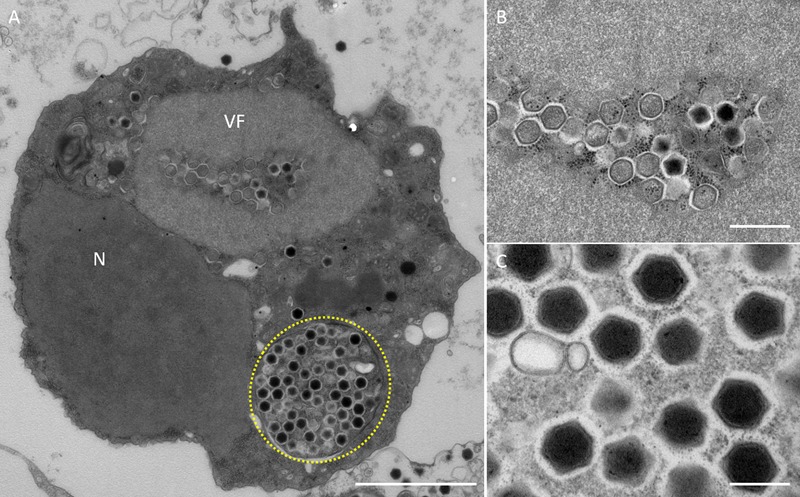
TEM images of ultrathin sections of amoeba cells infected with the new viruses isolated from the Ukawa River (named kashiwazakivirus). **(A)** TEM image of a typical virion factory (VF) in the cytoplasmic region of the amoeba cells. N indicates an amoeba cell nucleus. Yellow circle indicates membrane bag. Scale bar: 2 μm. **(B)** Enlarged image of virus particles in VF of panel **(A)**. Scale bar: 500 nm. **(C)** Enlarged image of viral particles in the amoeba cytoplasm. Scale bar: 200 nm.

**FIGURE 4:**
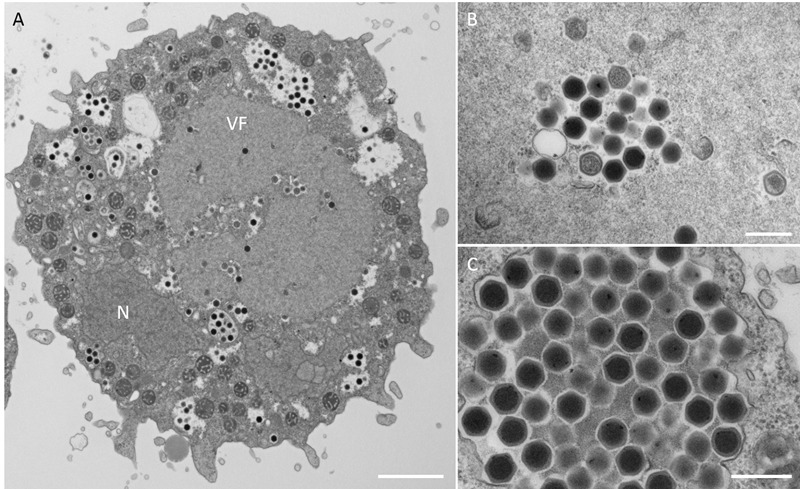
TEM images of ultrathin sections of amoeba cells infected with the new viruses isolated from the Tatakaigawa River in Kyoto (named kyotovirus). **(A)** TEM image of a typical virion factory (VF) in the cytoplasmic region of the amoeba cells. N indicates an amoeba cell nucleus. Scale bar: 2 μm. **(B)** Enlarged image of virus particles in VF. Scale bar: 400 nm. **(C)** Enlarged image of viral particles in the amoeba cytoplasm. Scale bar: 400 nm.

In this study, samples were collected from two sampling locations in Kashiwazaki City (small reservoir near Hokuto Town and the mouth of the Ukawa River) and one location in Kyoto (Tatakaigawa River in Uji City) (Figure 1). After inoculation of these samples into *A. castellanii* cells, we observed the virus-infected cells in a total of 15 wells where *A. castellanii* cells showed CPE (CPE-amoeba). The cytoplasm of these CPE-amoebas was observed by TEM. CPE-amoebas in one of three wells contained a sample from the small reservoir near Hokuto Town, one of five wells added with a sample from the mouth of the Ukawa River, and one of seven wells added with a sample from the Tatakaigawa River were selected, respectively. The cytoplasm of all CPE-amoebas was filled with numerous icosahedral virus particles with approximately 200 nm in diameter (Figure 2–4). Additionally, a “plant” of the virus, also known as “virion factory (VF)” replicating viral DNA, was formed in the amoeba cytoplasm (Figure 2A–4A), similarly to a previously reported *Marseilleviridae* VF (Boyer et al., 2009; Colson et al., 2013; Takemura, 2016). These viruses formed gigantic membrane bags containing numerous virus particles gathered in the cytoplasm (yellow circles in Figure 2A, 3A), where virus particles replicated in VF migrated into the bag and were released from the cell as previously described (Arantes et al., 2016). In CPE-amoebas infected with viruses isolated from a water sample in Kashiwazaki City, we observed that virus particles were lined up on the host cell surfaces (Supplementary Figure 1 and red arrows in Figure 2B). Based on these morphological characteristics, these viruses were considered as members of the family *Marseilleviridae*.

These viruses isolated from the 15 wells were tentatively named as “hokutoviruses 1–3,” which were viruses isolated from a small reservoir near “Hokuto” Town, “kashiwazakiviruses 1–5,” viruses isolated from the mouth of the Ukawa River near the “Kashiwazaki” Harbor, and “kyotoviruses 1–7,” viruses isolated from the Tatakaigawa River in Uji City, “Kyoto.” Later, we confirmed the classifications for most viruses, one of which was renamed.

### “Bunch” Formation of CPE-Amoeba Induced With Newly Isolated Viruses

**FIGURE 5:**
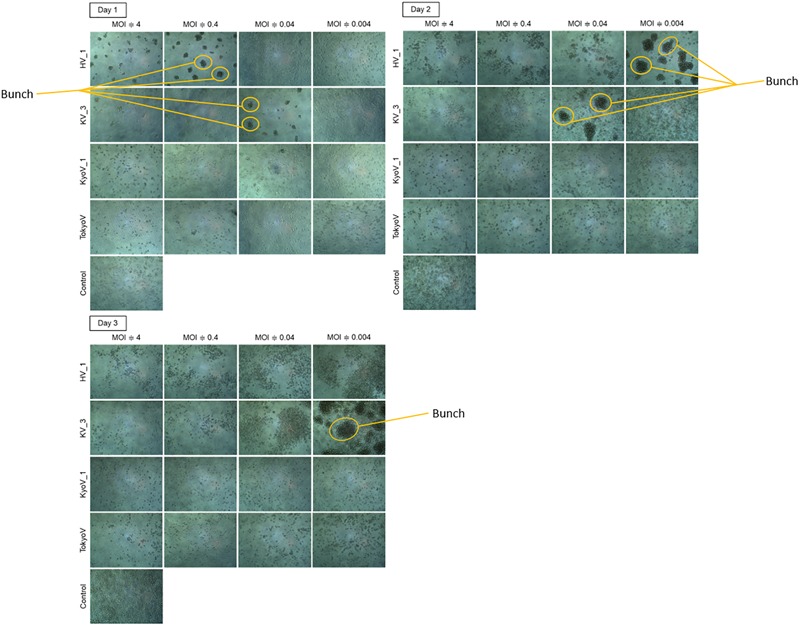
“Bunch” formation of the amoeba cells. Hokutovirus (HV) 1, kashiwazakivirus (KV) 3, kyotovirus (KyoV) 1, and tokyovirus (TokyoV) were inoculated into *A. castellanii* cells with four degrees of titers (MOIs are approximately 4, 0.4, 0.04, and 0.004 calculated using TCID_50_ values and *A. castellanii* cell count). Control: non-infected *A. castellanii* cells. Formed “bunch” are indicated by orange circles.

Amoeba cells infected with tupanvirus, one of the largest giant viruses belonging to the family *Mimiviridae* (Abrahão et al., 2018), gather with each other and form a “bunch,” which sometimes contains uninfected normal cells (Oliveira et al., 2019). Interestingly, the eight newly isolated hokutoviruses 1–3 and kashiwazakiviruses 1–5 induced amoeba cells to form a “bunch” likely in the early stages of infection (MOI: approximately 4). In amoeba cells infected with fewer viruses (MOI = 0.1), “bunch” formation was delayed but the bunch was ultimately than those observed in the former cases (Figure 5 and Supplementary Figure 2). This was not observed in the newly isolated kyotoviruses 1–7 and previously reported tokyovirus (Takemura, 2016) (Figure 5), suggesting that “bunch” formation is a unique property induced by hokutoviruses and kashiwazakiviruses.

### Design of MCP Primers of the Family *Marseilleviridae* in Several Lineages

**FIGURE 6:**
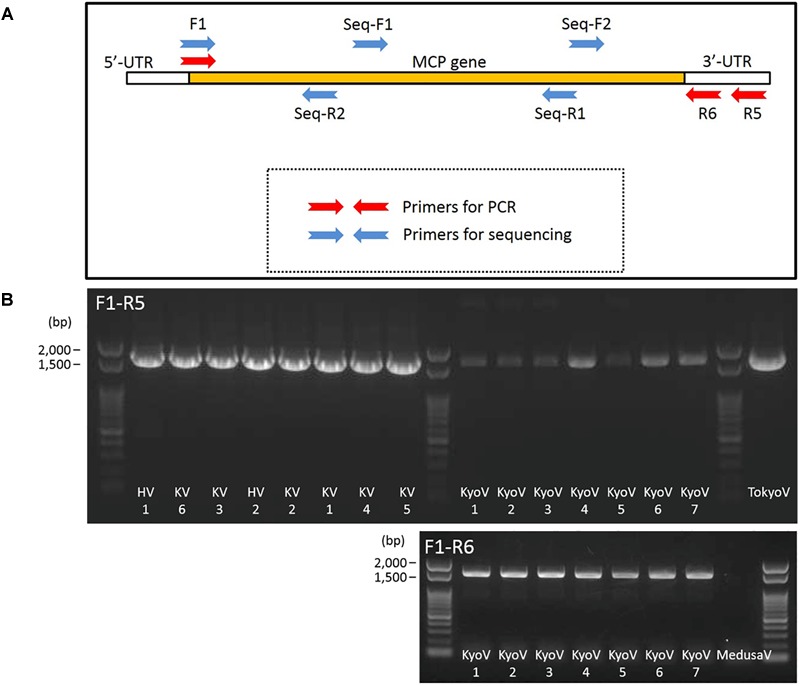
Primers for MCP genes of the family *Marseilleviridae*. **(A)** Design outline of primers for amplification of MCP genes of the family *Marseilleviridae* and 4 primers for sequencing. **(B)** PCR products of MCP genes of kashiwazakiviruses (KV), hokutoviruses (HV), and kyotoviruses (KyoV), using 2 sets of primers, F1-R5 and F1-R6. Tokyovirus (TokyoV) and medusavirus (MedusaV) were used as positive and negative controls, respectively.

To confirm the types of these isolated 15 marseillevirus-like viruses, we sequenced their MCP genes. To quickly identify MCP genes of members of the family *Marseilleviridae*, primers specific for the MCP genes that can be widely used for molecular phylogenetic analysis were designed. We attempted to design primers specific for homologous sequences by aligning the MCP genes of members of the family *Marseilleviridae* registered in the NCBI database (Supplementary Figure 3 and Figure 6A). Golden marseillevirus showed a low level of sequence similarity with MCP genes in other members of the family *Marseilleviridae*, which was not considered in our primer design. We confirmed that these primers did not amplify the MCP genes of *Mimivirus shirakomae* (Takemura et al., 2016) or medusavirus (Yoshikawa et al., 2019), suggesting that the primers can be used for rapid identification and screening of members of the family *Marseilleviridae* during giant virus isolation from various water and soil samples.

Consequently, MCP genes of the newly isolated viruses were successfully amplified by PCR using the designed primers. A primer set (F1 and R5) amplified the MCP genes of isolated hokutoviruses and kashiwazakiviruses, and the other primer set (F1 and R6) amplified MCP genes of isolated kyotoviruses and the previously isolated tokyovirus (Takemura, 2016) (Figure 6B).

### Molecular Phylogenetic Analysis of *Marseilleviridae* MCP and D5-Like Helicase-Primase Genes

**FIGURE 7:**
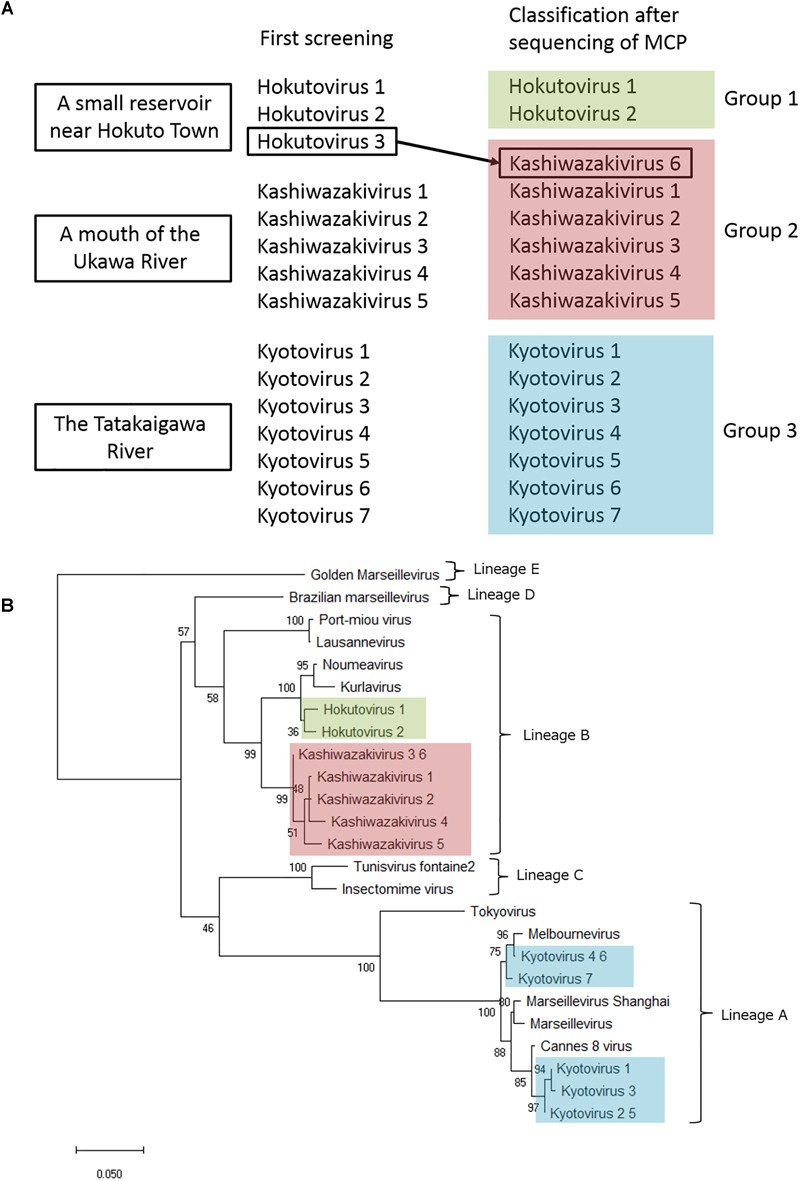
Analysis of MCP genes. **(A)** Three groups of newly isolated viruses. Hokutovirus 3, which was named after first screening, was re-named to kashiwazakivirus 6 after classification according to the phylogenetic analysis of MCP gene. **(B)** Molecular phylogenetic analysis of MCP genes of the family *Marseilleviridae*. An unrooted maximum likelihood tree of MCP was reconstructed using MEGA X software. A tree was reconstructed based on MCP gene nucleotide alignment (1,429 sites) derived from the full-length alignment. Numbers at the branch points denote percent bootstrap values. Color boxes indicate newly isolated viruses.

**FIGURE 8:**
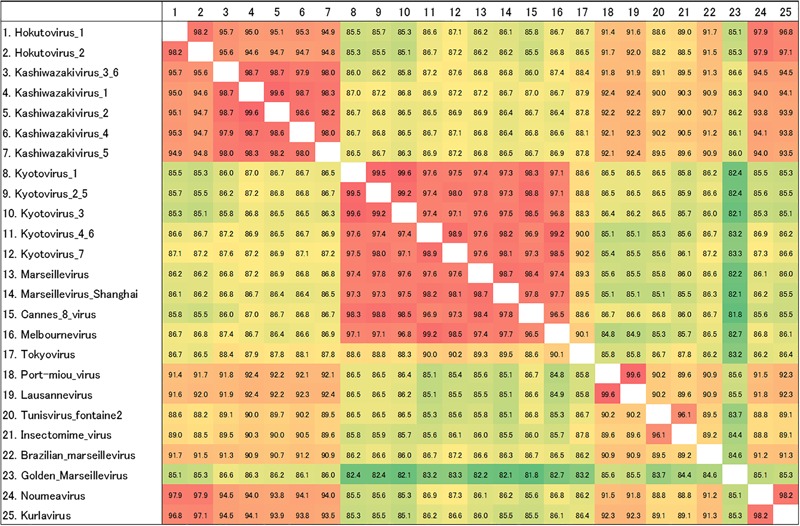
Pairwise sequence identity of MCP genes. The pairwise sequence identities are displayed in percentage. Color of each box corresponds to the order of values from red (high) to green (low). The numbers on the left of the species name match to the numbers on the first row.

Amplified full-length MCP genes of the newly isolated viruses were then subjected to sequence analysis. These newly determined MCP genes and those of previously described members of the family *Marseilleviridae* were aligned and a molecular phylogenetic tree of MCPs was reconstructed. Compared to previously reported members of the family *Marseilleviridae*, molecular phylogenetic analysis using the maximum likelihood method showed that all 15 isolated viruses contained slightly different MCP genes (Figure 7B and Supplementary Data 1). Among these, the MCP genes of kashiwazakivirus 3 and hokutovirus 3 (renamed as kashiwazakivirus 6 as described below, Figure 7A) had the same sequences, and those of kyotovirus 2 and 5, as well as those of kyotovirus 4 and 6, were also the same (Figure 7B), suggesting these viruses, kashiwazakiviruses 3 and 6, kyotoviruses 2 and 5, and kyotoviruses 4 and 6 are considered the same virus isolated twice. Additionally, these viruses also had the same sequences as partial D5-like helicase-primase genes (Supplementary Table 2 and Supplementary Figure 4). Three groups of newly isolated viruses were classified into three clades corresponding to two hokutoviruses (group 1), five kashiwazakiviruses and one hokutovirus (group 2), and seven kyotoviruses (group 3) (Figure 7A). Accordingly, we renamed hokutovirus 3 classified into group 2 as “kashiwazakivirus 6” (Figure 7A). Group 1 was more closely related to lineage B, which includes noumeavirus and kurlavirus (Figure 7B). Six kashiwazakiviruses, which are in group 2, appeared to form an independent group more closely related to lineage B and group 1 (Figure 7B). In contrast, group 3 belongs to lineage A (Figure 7B). Pairwise sequence identity of the MCP genes of each virus from the family *Marseilleviridae* supported the classification described above (Figure 8). Similar phylogenetic trees and grouping of newly isolated marseilleviruses were reconstructed using partial sequences of D5-like helicase-primase genes (Supplementary Figure 4 and Supplementary Data 2).

Interestingly, two viruses belonging to independent groups possessed the same MCP genes as described above. Kashiwazakivirus 3 and 6 (former name hokutovirus 3) were isolated from the Ukawa River and a small reservoir near Hokuto Town, respectively. These results suggest that several giant viruses, which have slightly different MCP genes, can be isolated from a single location, and that the same (putatively identical or very similar) giant viruses (i.e., same MCP) can be isolated from different places. Although seven kyotoviruses were classified into lineage A, they slightly differed from each other (Figure 7B, 8). In detail, the MCP genes of kyotovirus 1, 2, 3, and 5 were closely related to those of Cannes 8 virus, while MCP genes of kyotovirus 4, 6, and 7 were relatively close to melbournevirus (Figure 7B, 8). Among these, kyotovirus 2 and 5 and kyotovirus 4 and 6 possessed the same MCP. These viruses were also found to share the same partial sequences of D5-like helicase-primase genes (Supplementary Figure 4). Thus, we newly isolated 15 viruses as members of the family *Marseilleviridae*, including 12 viruses with different MCP genes, from aquatic environments in Japan, in addition to a single tokyovirus (Figure 7).

## Discussion

In this study, we successfully isolated at least 12 new viruses with different MCP genes in the family *Marseilleviridae* from three sampling locations in Japan. MCP is one of the core genes of NCLDVs and thus has been used for molecular phylogenetic analysis of giant viruses as a part of concatenated genes (Thomas et al., 2011; Dornas et al., 2016; Dos Santos et al., 2016; Chatterjee and Kondabagil, 2017; Boratto et al., 2018). Using the MCP gene alone, we reconstructed a molecular phylogenetic tree. This method can be used to rapidly and tentatively classify members of the family *Marseilleviridae* more clearly than by using concatenated genes as reported previously (Dornas et al., 2016). To test their MCP genes, we designed and used two sets of primers (F1-R5 and F1-R6) to amplify the MCP genes of the family *Marseilleviridae*, which are useful for detecting MCP genes in lineages A and B. These two lineages are not closely related, suggesting that the primers can also be applied to detect MCP genes of other lineages of the family *Marseilleviridae*. Thus, this method using MCP gene can be used to rapidly and tentatively classify each member of the family *Marseilleviridae* more easily.

Members of the family *Marseilleviridae* have been isolated from various aquatic environments worldwide. However, tokyovirus has been the unique member of the family *Marseilleviridae* in East Asia and South-East Asia, which was isolated from the Arakawa River in Tokyo (Takemura, 2016). Although a complete genome sequence of the putative isolate from China, *Marseillevirus shanghai*, recently became available in NCBI, Asia has remained a “blank area” of the family *Marseilleviridae* except for kurlavirus discovered in India (Chatterjee and Kondabagil, 2017) and tokyovirus. Japan is well-known to be rich in aquatic environments with a large number of rivers, ponds, and spring waters, as well as high rainfall throughout the year. Therefore, we attempted to isolate new giant viruses from Japanese aquatic environments, and successfully isolated 12 or 15 marseilleviruses in this study. Molecular phylogenetic analysis based on sequence analysis of the MCP genes clearly showed that 12 new marseilleviruses with different MCP genes could be classified into three virus groups of the family *Marseilleviridae*. One group, which included 5 kyotoviruses, was suggested to belong to lineage A, which includes *Marseillevirus marseillevirus*, melbournevirus, Cannes 8 virus, senegalvirus, tokyovirus, and *Marseillevirus shanghai*. Regarding the MCP genes, our results suggest that kyotoviruses are highly similar to melbournevirus and Cannes 8 virus. The other two groups, kashiwazakivirus and hokutovirus, were not as highly similar to kyotovirus. Hokutovirus contains MCP genes highly similar to lineage B (particularly kurlavirus and noumeavirus). Kashiwazakivirus may form a new subgroup of lineage B (Figure 7B).

We observed two notable results. First, these multiple viruses were isolated from a single water sample in a single sampling location. Second, two kashiwazakiviruses containing exactly the same MCP genes (kashiwazakiviruses 3 and 6) were isolated from two different sampling locations (Figure 7A). Thus, the family *Marseilleviridae* has more diversity in local aquatic environments than originally considered, i.e., at least 2 lineages, A and B (Figure 7), inhabit in small islands in Japan, and several members of the family *Marseilleviridae* can be isolated from a single sampling location.

In addition to MCP gene variations, each virus showed differences in phenotype. Amoeba cells infected with kashiwazakivirus or hokutovirus showed that CPE-amoebas were not only rounded but also formed “bunches,” as observed for tupanviruses (Oliveira et al., 2019), suggesting that “bunch” formation is a relatively common property in some giant viruses. In “bunch” formation of tupanvirus-infected amoeba cells, a mannose-binding protein (MBP) expressed on the amoeba cell membrane was suggested to be involved in intercellular adhesions (Oliveira et al., 2019). MBP, a type of lectin, is important for intercellular adhesion and intracellular communication, and is also encoded in tupanvirus genomes (Abrahão et al., 2018; Oliveira et al., 2019). For tupanvirus, adding mannose to the culture medium to inhibit intracellular adhesion by MBP resulted in inhibition of “bunch” formation (Oliveira et al., 2019). Although no homologous gene of tupanvirus MBP were identified in members of the family *Marseilleviridae*, we consider that similar mechanisms exist in “bunch” formation in the infected amoeba cells of kashiwazakivirus and hokutovirus. Interestingly, “bunch” formation was not observed in tokyovirus- or kyotoviruses-infected amoeba cells, suggesting that this process in the family *Marseilleviridae* is a specific feature in lineage B including kashiwazakivirus and its close relatives. The relationship between “bunch” formation and viral particles lining up on the host cell surfaces (Supplementary Figure 1 and Figure 2B) remains unclear.

CPE-amoebas were observed in several wells of the 96-well plates inoculated with a water sample from a single sampling location. Viruses causing amoeba cell CPE in each well had slightly different MCP genes. In future studies of giant viruses or NCLDVs, there may be several different “genotypes” of viruses in a single sample location, providing additional opportunities to identify new viruses in the future. Many viruses are present in a few milliliters of water, and further studies of the local diversity of the NCLDV family including the family *Marseilleviridae* are necessary. Furthermore, the mechanism of “bunch” formation in addition to virus–host interactions should be examined to determine the history of evolution of the family *Marseilleviridae* through comparative analysis of the various genes using isolated viruses, including more detailed molecular biological and biochemical studies.

## Data Availability

Full-length sequences of MCP genes of hokutoviruses, kashiwazakiviruses, and kyotoviruses have been submitted to DDBJ (accession number: hokutovirus 1, LC477072; hokutovirus 2, LC477073; kashiwazakivirus 1, LC477074; kashiwazakivirus 2, LC477075; kashiwazakivirus 3, LC477076; kashiwazakivirus 4, LC477077; kashiwazakivirus 5, LC477078; kashiwazakivirus 6, LC477079; kyotovirus 1, LC477080; kyotovirus 2, LC477081; kyotovirus 3, LC477082; kyotovirus 4, LC477083; kyotovirus 5, LC477084; kyotovirus 6, LC477085; kyotovirus7, LC477086).

## Author Contributions

KA and RH isolated the hokutoviruses and kashiwazakiviruses. KA and MT analyzed “bunch” formation. HO isolated kyotoviruses. KA and KM performed TEM analysis of hokutoviruses and kashiwazakiviruses. MT designed the *Marseilleviridae* primers for MCP genes, sequence analysis, and molecular phylogenetic analysis of MCP genes. KA and MA performed heatmap analysis. KA and KS performed sequence and phylogenetic analysis of D5-like helicase-primase genes. MA and MT designed the research. KA and MT wrote the initial manuscript. All authors contributed to the finalization of the manuscript.

## Conflict of Interest Statement

The authors declare that the research was conducted in the absence of any commercial or financial relationships that could be construed as a potential conflict of interest.

## Footnotes

1 https://www.ncbi.nlm.nih.gov/
