# Supplementary material for: Fifteen Marseilleviruses Newly Isolated From Three Water Samples in Japan Reveal Local Diversity of Marseilleviridae
Source: Front Microbiol. 2019 May 24;10:1152. doi: 10.3389/fmicb.2019.01152 (PMC6543897; doi:10.3389/fmicb.2019.01152)
Supplement: Supplementary file 1 [file Data_Sheet_1.PDF]

Supplementary Material

1. Supplementary Methods

Design of primers for D5-like helicase-primase gene of the family *Marseilleviridae*

To amplify D5-like helicase-primase genes of the family *Marseilleviridae*, we designed primers as described previously (Popgeorgiev et al., 2013). Two forward primers, Helicase-F1 and Helicase-F2 (20 bases each), were designed; 5′- GAYCCAAAYKCSCAGCTKAC-3′ and 5′-AGACCCAAACTCGCAGCTTA-3′ (Supplementary table 2). Two reverse primers, Helicase-R1 and Helicase-R2 (20 bases each), were designed; 5′- AARATDCCRAGTTTSARGTC-3′ and 5′-CCGGAAGATTCCGAGTTTCA-3′ (Supplementary table 2). Helicase-F1 and Helicase-R1 were used for PCR of D5-like helicase-primase genes of hokutovirus and kashiwazakivirus. Helicase-F2 and Helicase-R2 were used for PCR of D5-like helicase-primase genes of kyotovirus. PCR products (159 bases) corresponded to a region ranging from 1,444 to 1,602 of the D5-like helicase-primase gene of *Marseillvirus marseillevirus*. Abbreviations of mixed bases are described in the section of Design of primers for MCP gene of the family *Marseilleviridae*.

Sequence and phylogenetic analyses of D5-like helicase-primase genes

After virus cloning, the genomic DNA of each hokutovirus, kashiwazakivirus, and kyotovirus was extracted from viral particles as described in the section of Sequence analysis of MCP genes. Partial D5-like helicase-primase genes were then amplified by PCR using extracted genomic DNA as a template. Amplified partial D5-like helicase-primase genes of these viruses were sequenced using 4 primers Helicase-F1, Helicase-F2, Helicase-R1, and Helicase-R2 for sequencing (Supplementary table 2). Capillary sequences were performed by Fasmac Co., Ltd. Partial sequences of each D5-like helicase-primase are shown in Supplementary data 2. Nucleotide sequences of D5-like helicase-primase genes of members of the family *Marseilleviridae*, *Marseillevirus marseillevirus*, lausannevirus, melbournevirus, senegalvirus, fontaine Saint-Charles virus, tunisvirus, insectomime virus, Cannes 8 virus, Port-Miou virus, tokyovirus, *Marseillevirus shanghai*, Brazilian marseillevirus, golden marseillevirus, noumeavirus, and kurlavirus were obtained from the NCBI nucleotide sequence database (https://www.ncbi.nlm.nih.gov/). Partial sequences and newly sequenced partial D5-like helicase-primase sequences of all hokutoviruses, kashiwazakiviruses, and kyotoviruses were aligned in the same manner as MCP genes. To reconstitute a maximum-likelihood tree, we estimated branch support with 1,000 bootstrap replications and used the K2 model as the substitution model with invariant sites (I). Gaped sites were removed before calculation, and 159 sites were used.

2. Supplementary Tables

Table S1. Primers used for sequence analysis of MCP gene.

Primer name	Nucleotide sequence
Seq-F1	5′-GATHAACTACTGTCTYCGCAG-3′
Seq-F2	5′-CCACGCRTACTCKATYTCKC-3′
Seq-R1	5′-RTTGATKGTCGTCTCRTTGAC-3′
Seq-R2	5′-CACRGAMGCRGAGAAYTCDK-3′

Table S2. Primers used for PCR and sequence analysis of D5-like helicase-primase gene.

Primer name	Nucleotide sequence
Helicase-F1	5′-GAYCCAAAYKCSCAGCTKAC-3′
Helicase-R1	5′-AARATDCCRAGTTTSARGTC-3′
Helicase-F2	5′-AGACCCAAACTCGCAGCTTA-3′
Helicase-R2	5′-CCGGAAGATTCCGAGTTTCA-3′

3. Supplementary Figures

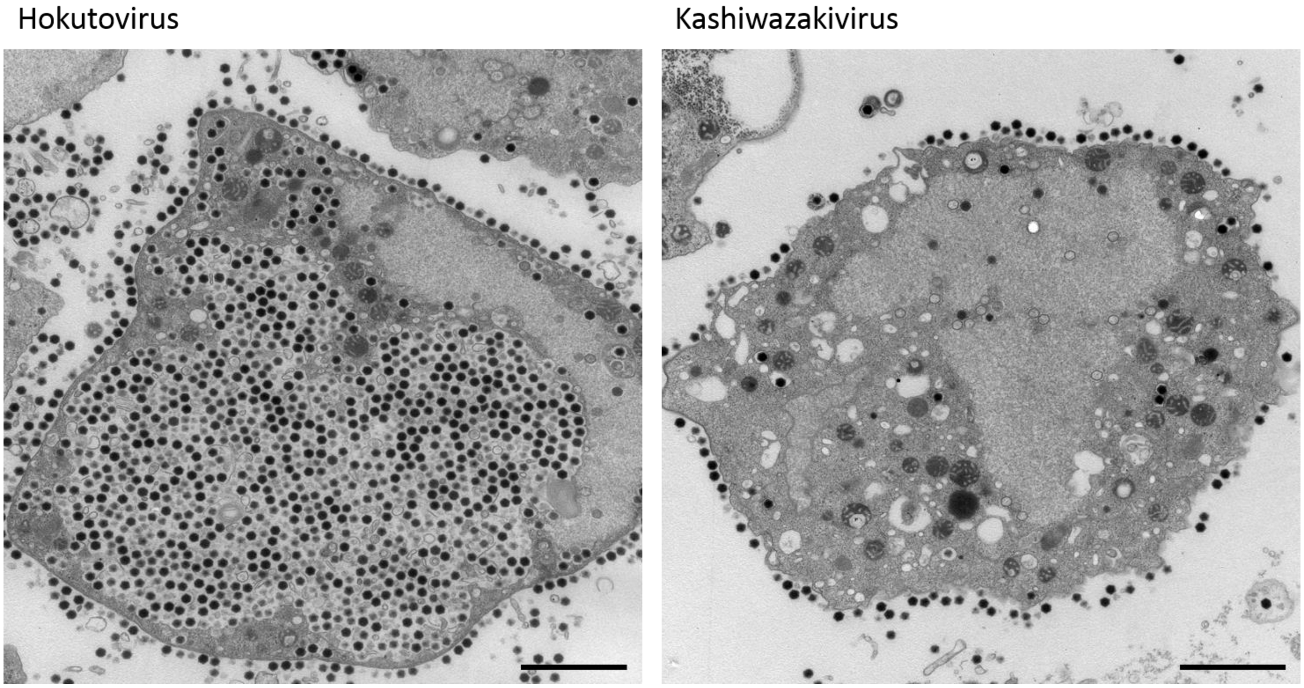

Supplementary Figure 1. TEM images of ultrathin sections of amoeba cells infected with new viruses isolated from a small reservoir near Hokuto Town (named hokutovirus) and from the Ukawa River (named kashiwazakivirus). Scale bars: 2 µm.

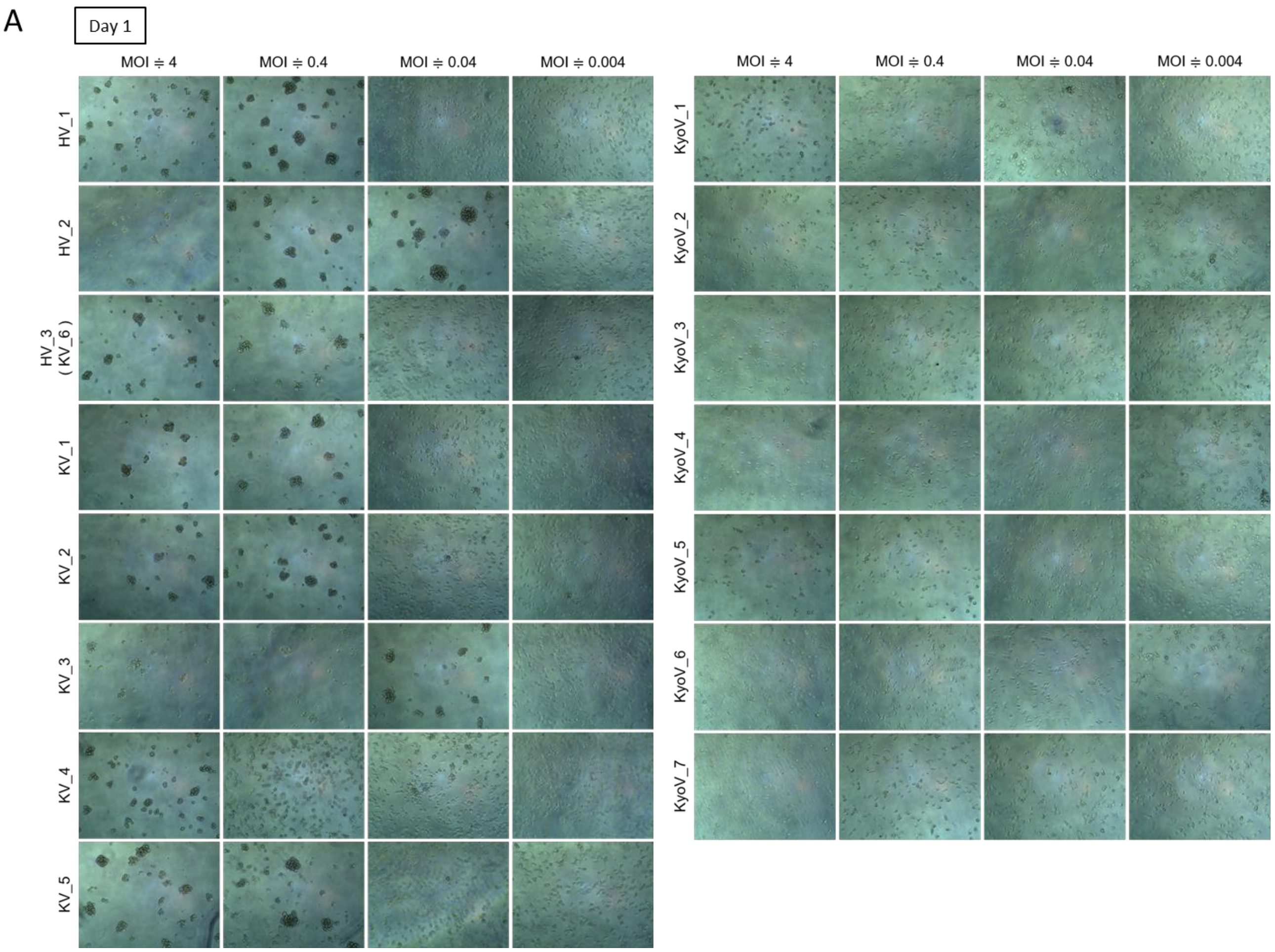

Supplementary Figure 2. “Bunch” formation of amoeba cells. Hokutovirus (HV) 1–3 (later named as kashiwazakivirus 6), kashiwazakivirus (KV) 1–5, and kyotovirus (KyoV) 1–7, were inoculated into *A. castellanii* cells with four degrees of titers (MOIs are approximately 4, 0.4, 0.04, and 0.004 calculated using $TCID_{50}$ values and *A. castellanii* cell count). (A) One day after infection.

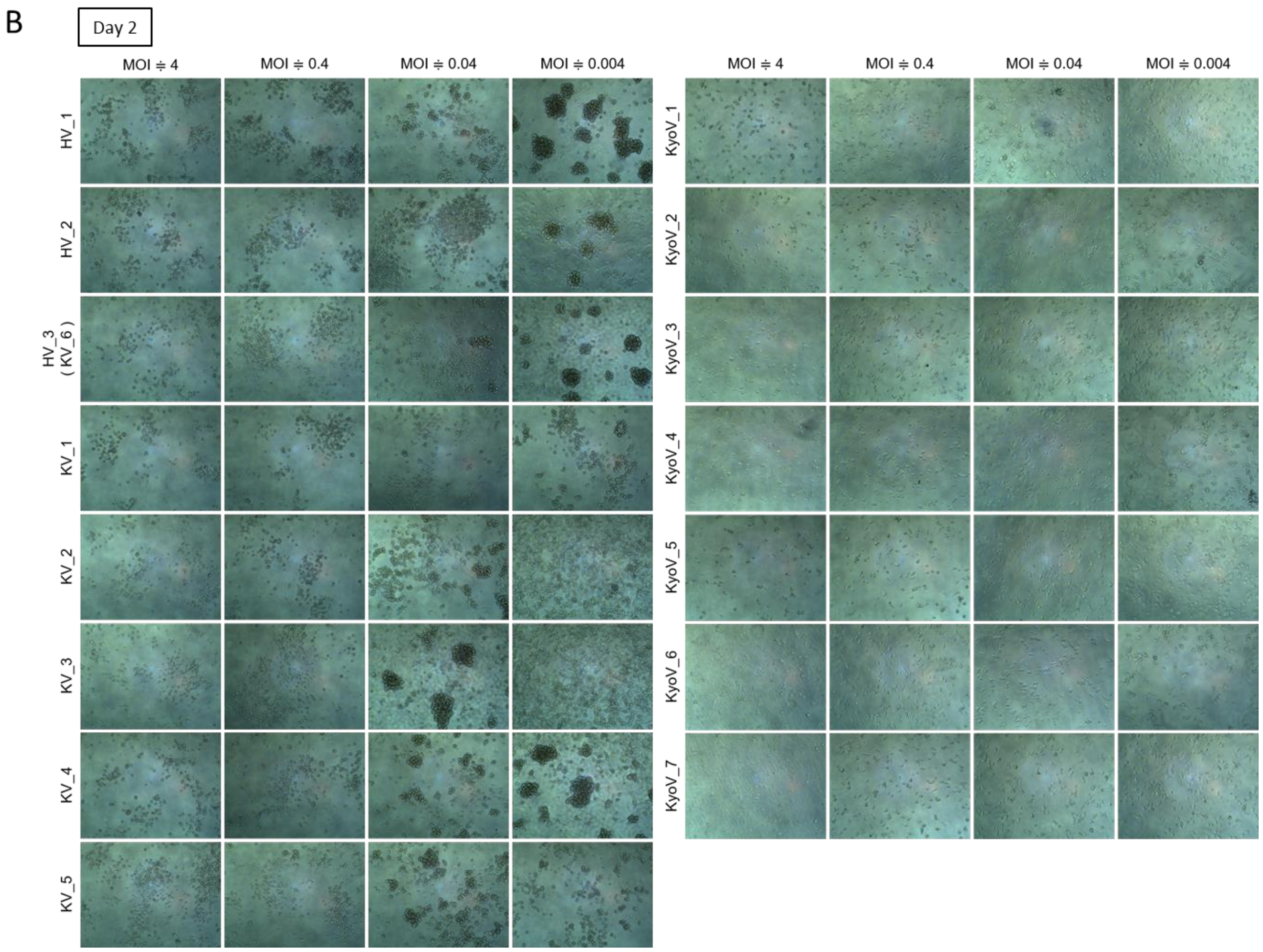

Supplementary Figure 2. (B) Two days after infection.

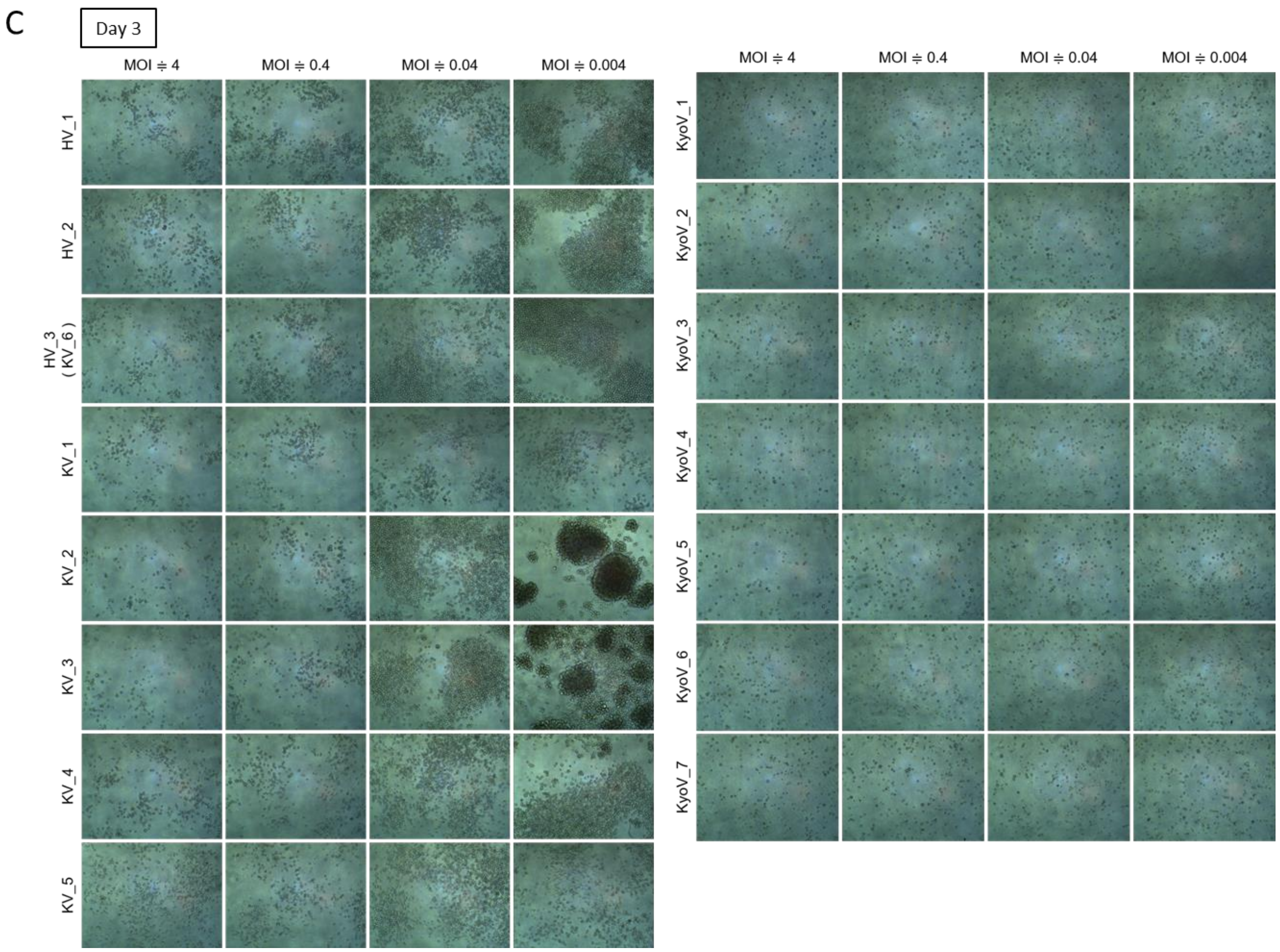

Supplementary Figure 2. (C) Three days after infection.

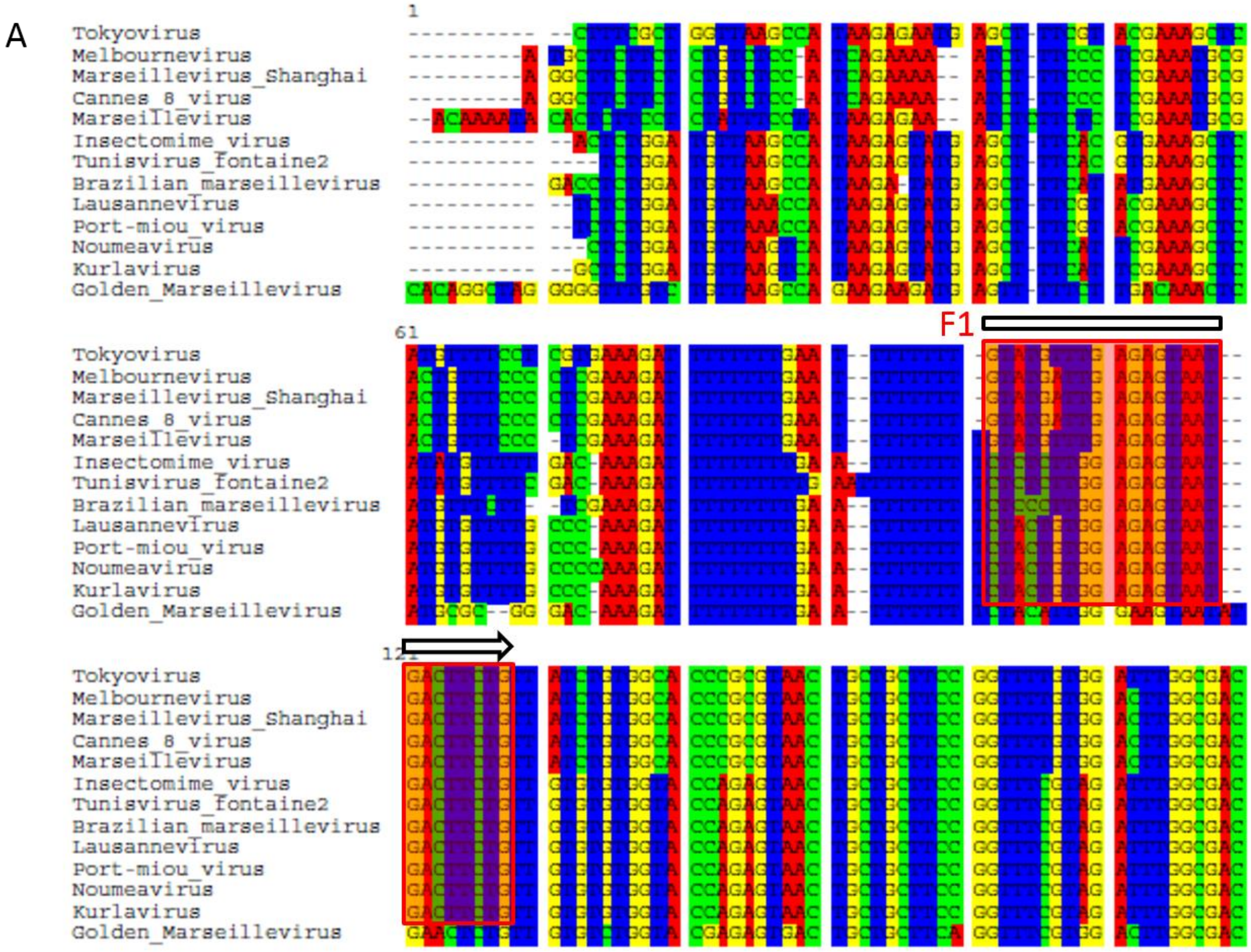

Supplementary Figure 3. Sites of PCR primers for MCP genes of the family *Marseilleviridae*. (A) MCP genes of known 13 members of the family *Marseilleviridae* were aligned using the ClustalW program implemented in MEGA X software (ver.10.0.3) with default parameters. Site of primer F1 is indicated by white arrow and red box.

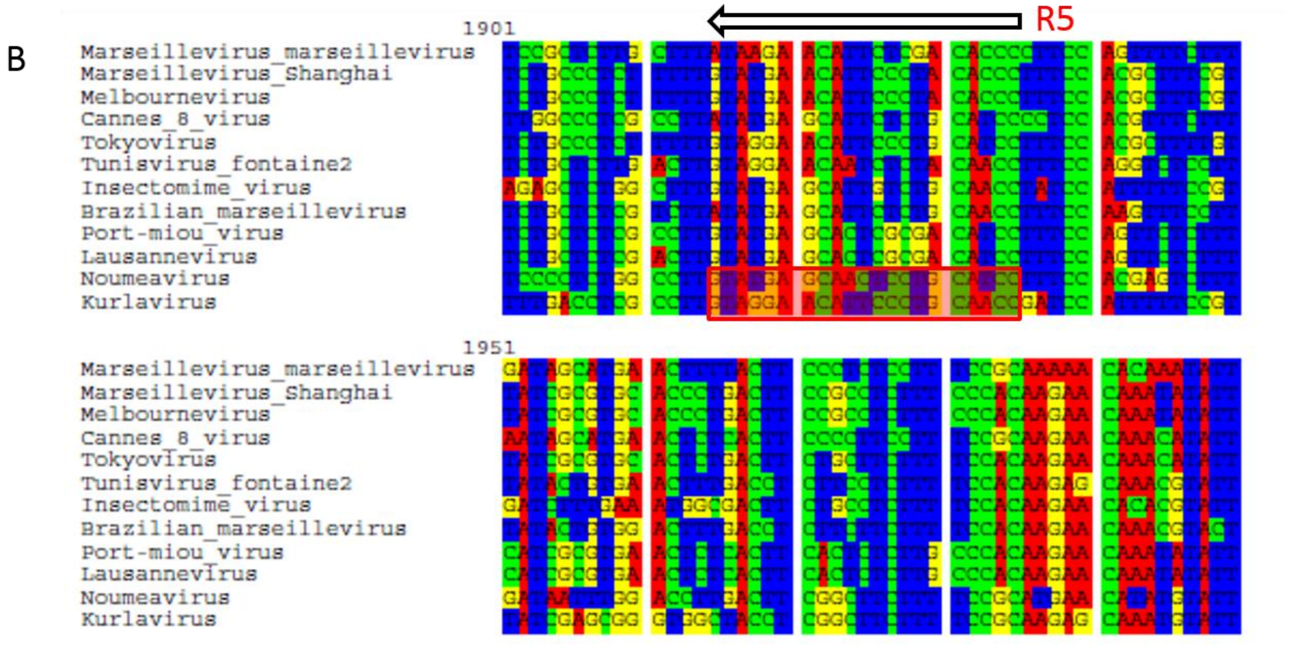

Supplementary Figure 3. (B) MCP genes of known 12 members of the family *Marseilleviridae* were aligned as described in panel A. Site of primer R5 is indicated by white arrow and red box.

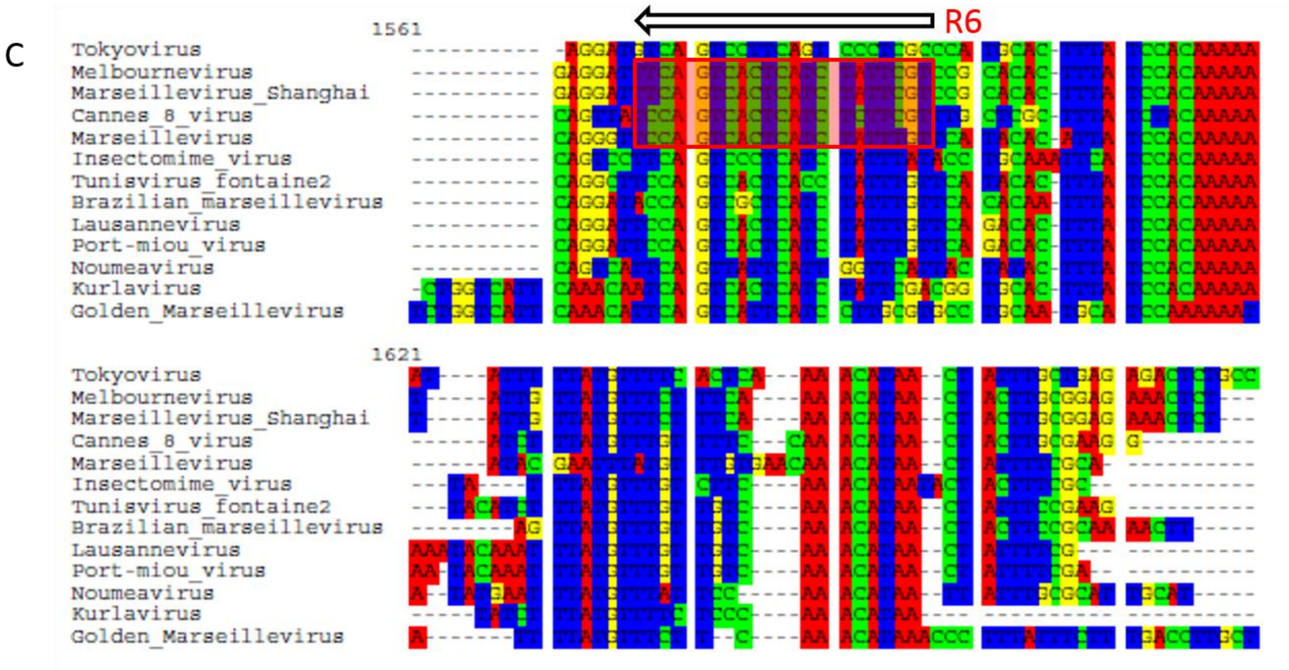

Supplementary Figure 3. (C) MCP genes of known 13 members of the family *Marseilleviridae* aligned as described in panel A. Site of primer R6 is indicated by white arrow and red box.

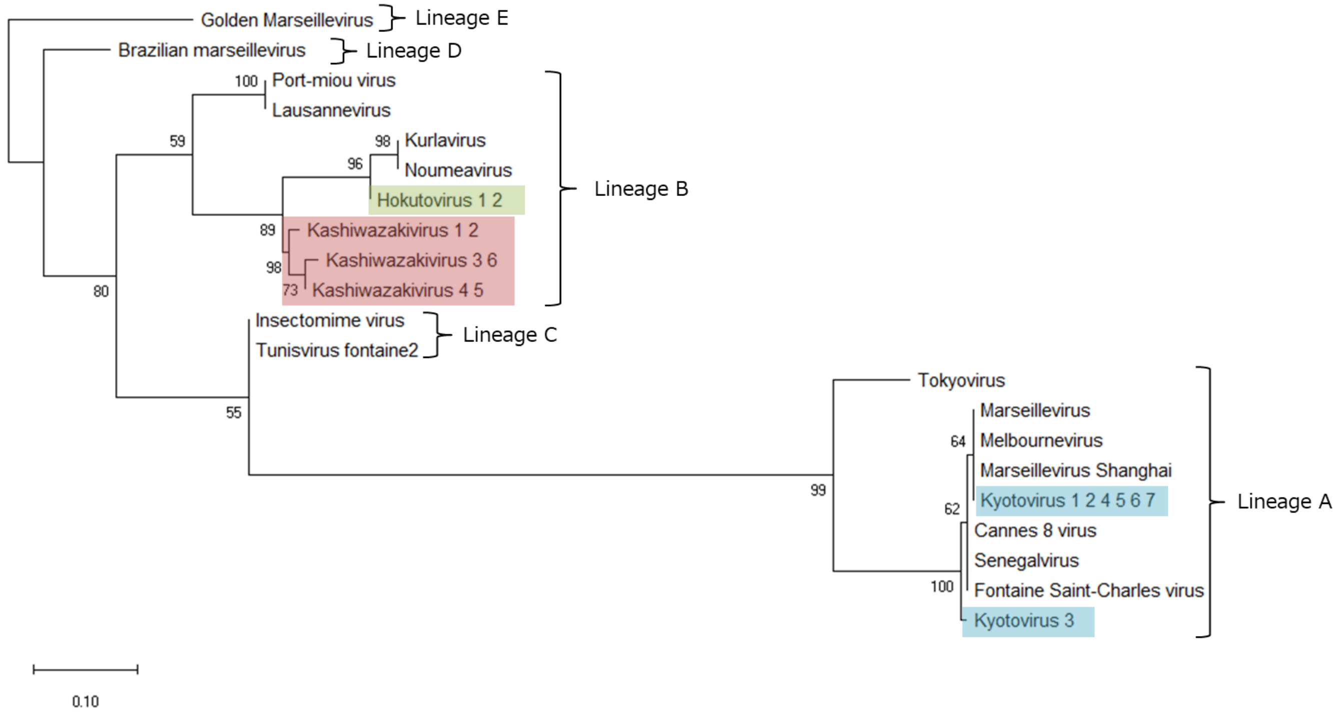

Supplementary Figure 4. Molecular phylogenetic analysis of partial D5-like helicase-primase genes of the family *Marseilleviridae*. An unrooted maximum likelihood tree was reconstructed using MEGA X software. A tree was reconstructed based on nucleotide alignment (159 sites) derived from partial alignment. Numbers at the branch points denote percent bootstrap values. Color boxes indicate newly isolated viruses.

4. Supplementary Data

Supplementary Data 1. Full-length sequences of MCP genes of newly isolated 15 viruses.

>Hokutovirus_1
ATGACTTCTGTTGTGTGTGGTACCAGAGTAACTGCTGCTTCCGGTTTCGTAGATTTGGCGACCTTCTCCGACTTGGAGGCGTATCTCTATGGT
GGTTGCTCGGCAGTCACCTATTTTGTGCGTGCCATCAAAAAGGCCAATTGGTTCTCTTTCCTCCCCGTTGTTCTCCGAAACATTTCGGGTCTT
CCCGGCTTCGGTAATGAATTCTCCGCGTCTGTGAATCGCTCCGGTGATTACGTCCTCAACACCTGGCTTCGCGTCCGTCTTCCTCTCATTGCT
ATCCGTCCGACCAATGCTGGTGGTGCCATTAACGCTAACGCCACCATCCGCTGGACCAGGAACTTTATGCACAATCTTGTTGAAAAGATTAAC
ATCACTTTCAACGATCTGATTGTTCACGAGTTTGACAGCTATTGGTTCGACTTTAACTCTCAGTTCAATATTGATGCTTCCAAGCGCGTCGGT
TACAGGAACATGATTGGAGACATTCCCGCCATGATTAACCCTGTGACGACCGGCAACCCCCTCGGCACCGGCGAATTCTTCAACCTTCCTATC
CCCCTCTTTTATTCAGAGGACTCGGGTCTGGCCCTTGCCGTTTCAGCCCTTCCCTTCAACGACATCAAGATTAACTACTGTCTCCGCAGGTGG
CAGGATCTTCTCGTCCTGAACGTGGGTGTTGGCGCCAACCCTCCCACTCTCGACGATGTTGTTCAGGTGAGCTACGATGCCACCTTCACCCTG
ATTTACAGCTCCAACCCTCCCGCCATCACCAATGTCGAGACTTGGTGCCACTACGCCGTCGTGCACAATGACGAGCGTGTCAAGATGGGTAAG
AATCCCCGTGATATGGTCATCAAGCAGGTTCAGAAGGTCAATGAGACGACCATCAACCTTTCGCAGCTCAACGCTCTCATCCCGATTGATATC
CGTATCTCTCACGCCGTTGTTGGATACTTCTACGCCATCCGAAACAGCTCCACTCCCGGCGAATGGTCCAACTACACTACCGAGCCCGCCTAT
GCCGGTCTTGACCCGCTTGAGGCTGCCCAGCTTGTTTACGAGTCGACTGCCCGTGTCAGCAACGGTTCCGACTATTACAGTCTTGTTGTGCCG
TGGTACTGGCACAAGTCGATCCCCGAGGAGACCGGCTACCACGCGTACTCGTACTCGCTGGAGACTTTCGCCTCTGACCCCAAGGGTTCGACC
AATTATTCCAAGCTCACCAATGTGTCGAACCAGTACGTTCCTTCGACCGCCGCCGTCAACGCCTCTGCTGGTGTGACCAACACCGGTATTCCG
ATTCCCTCAGCCACCAATCCTGCTGTGACTCAGCAGAACCAGACTTTCCAGCATATCTTCCGAGTCCTTAATTTCAACGTGCTTAGATTGTCG
GGAGGCAGCTTGGGTCTTCCCATATTGTGA
>Hokutovirus_2
ATGACTTCTGTTGTGTGTGGTACCAGAGTAACTGCTGCTTCCGGTTTCGTAGATTTGGCGACCTTCTCTGACTTGGAGGCGTATCTCTATGGT
GGTTGCTCGGCAGTCACCTATTTCGTGCGTGCCATCAAAAAGGCTAATTGGTTCTCCTTCCTTCCCGTTGTTCTCCGAAATATTTCGGGTCTT
CCCGGCTTCGGTAATGAATTCTCCGCGTCTGTGAATCGCTCCGGTGATTACGTCCTCAACACCTGGCTGCGCGTCCGTCTCCCTCTGATTGCT
ATTCGCCCGACCAATGCTGGTGGCGCCATCAATGCTAACGCCACCATCCGCTGGACCCGAAACTTTATGCACAATCTTGTTGAAAAGGTCAAT
ATCACCTTCAATGATCTGATTGTTCATGAGTTTGACAGCTACTGGTTCGACTTTAACTCTCAGTTCAATATTGATGCTTCCAAGCGCGTCGGT
TACAGGAACATGATTGGAGACATTCCCGCCATGATTAACCCTGTGACGACCGGCAACCCCCTCGGCACCGGCGAATTCTTCAACCTTCCTATC
CCCCTCTTTTATTCAGAGGACTCGGGTCTGGCCCTTGCCGTTTCAGCCCTTCCCTTCAACGACATCAAGATTAACTACTGTCTCCGCAGGTGG
CAGGATCTTCTCGTCCTGAACGTGGGTGTTGGCGCCAACCCTCCCACTCTCGACGATGTTGTTCAGGTGAGCTACGATGCCACCTTCACCCTG
ATTTACAGCTCCAACCCTCCCGCCATCACCAATGTCGAGACTTGGTGCCACTACGCCGTCGTGCACAATGACGAGCGTGTCAAGATGGGTAAG
AATCCCCGTGATATGGTCATCAAGCAGGTTCAGAAGGTCAATGAGACGACCATCAACCTTTCGCAGCTCAACGCTCTCATCCCGATTGATATC
CGTATCTCTCACGCCGTTGTTGGATACTTCTACGCCATCCGAAACAGCTCCACTCCCGGCGAATGGTCCAACTACACCACCGAGCCCGCCTAT
GCCGGTCTTGACCCGCTTGAGGCTGCCCAGCTTGTTTACGAGTCGACTGCCCGTGTCAGCAACGGTTCCGACTATTACAGTCTTGTTGTGCCG
TGGTACTGGCACAAGTCGATCCCCGAGGAGACCGGCTACCACGCGTACTCGTACTCGCTGGAGACTTTCGCCTCTGACCCCAAGGGTTCGACC
AATTATTCCAAGCTCACCAATGTGTCGAACCAGTACGTTCCTTCGACCGCCGCCGTCAACGCCTCTGCTGGTGTGACCAACACCGGTATTCCG
ATTCCCTCAGCCACCAATCCTGCTGTGACTCAGCAGAACCAGACTTTCCAGCATATCTTCCGAGTCCTTAATTTCAACGTGCTTAGATTGTCG
GGAGGCAGCTTGGGTCTTCCCATATTGTAG
>Kashiwazakivirus_3_6
ATGACTTCTGTTGTGTGTGGTACCAGAGTAACTGCTGCTTCCGGTTTCGTAGATTTGGCGACCTTCTCCGACTTGGAGGCGTACCTTTATGGT
GGTTGCTCGGCGGTCACCTATTTCGTGCGTGCCATCAAAAAGGCTAATTGGTTCTCTTTCCTCCCCGTTGTTCTCCGAAACATTTCGGGTCTT
CCCGGTTTCGGTAACGAATTCTCCGCGTCCGTGAATCGTTCCGGCGATTACGTCCTCAACACCTGGCTTCGCGTCCGTCTTCCTCTCATTGCT
ATCCGTCCGACCAATGCTGGTGGTGCCATCAACGCTAATGCCACCATCCGCTGGACCCGAAACTTCATGCACAATCTTGTTGAAAAGATCAAC
ATCACCTTCAATGATCTGATTGTCCACGAGTTTGACAGCTACTGGTTCGACTTTAACTCCCAGTTCAACATCGATGCTTCCAAGCGCGTCGGT
TACAGGAACATGATCGGAGACATTCCCGCCATGATTAACCCCGTGACGACCGGCAACCCCCTCGGCACCGGCGAATTCTTCAATCTTCCCATC
CCCCTCTTCTATTCCGAGGACTCGGGTCTGGCTCTTGCCGTCTCTGCCCTTCCTTTCAACGACATCAAGATCAACTACTGTCTCCGCAGGTGG

CAGGATCTTCTCGTCCTGAACGTGGGTGTTGGCGCCAACCCTCCCACTTTCGACGATGTTGTTCAGGTGAGCTACGACGCCACCTTCACCCTC
ATTTACAGCTCGAACGCTCCCGCCATCACCAATGTCGAGACTTGGTGCCACTACGCCGTTGTGCACAATGACGAGCGTGTCAAGATGGGTAAG
AATCCCCGTGATATGGTCATCAAGCAGGTTCAAAAGGTCAATGAGACGACCATCAATCTTTCGCAGCTCAACGCTCTCATCCCGATTGATATC
CGTATCTCTCACGCCGTCGTTGGCTATTTCTACGCCATCAGGAACAGCTCCACTCCCGGCGAATGGTCCAACTACACCACCGAGCCCGCCTAT
GCCGGTCTTGACCCGCTTGAGGCTGCCCAGCTTGTGTACGAGTCGACTGCCCGTGTCAGCAATGGCTCTGATTATTACAGCCTTGTTGTGCCG
TGGTACTGGCACAAGTCGATCCCCGAGGAGACCGGCTACCACGCGTACTCGTACTCTCTGGAGACTTTCGCCTCTGATCCCAAGGGTTCGACC
AATTATTCCAAGCTCACCAATGTGTCGAACCAGTACGTTCCTTCGACCGCTGCCGTCAACGCCTCTGCTGGTGTGACCAACACCGGTATTCCG
ATTCCCTCGGCCACCAATCCTGCTGTGACTCAGCAGAACCAGACTTTCCAGCACATCTTCCGAGTCCTTAATTTCAACGTGCTTAGATTGTCG
GGAGGCAGCTTGGGTCTTCCCATATTGTAG
>Kashiwazakivirus_1
ATGACTTCTGTTGTGTGTGGTACCAGAGTAACTGCTGCTTCCGGTTTCGTAGATTTGGCGACCTTCTCCGACTTGGAGGCGTACCTTTATGGT
GGTTGCTCGGCGGTCACCTATTTCGTGCGTGCCATCAAAAAGGCTAATTGGTTCTCTTTCCTCCCCGTTGTTCTCCGAAACATTTCGGGTCTT
CCCGGTTTCGGTAACGAATTCTCCGCGTCCGTGAATCGTTCCGGCGATTACGTCCTCAACACCTGGCTTCGCGTCCGTCTTCCTCTCATTGCT
ATCCGTCCGACCAATGCTGGTGGTGCCATCAACGCTAATGCCACCATCCGCTGGACCCGAAACTTCATGCACAATCTTGTTGAAAAGATCAAC
ATCACCTTCAATGATCTGATTGTCCACGAGTTTGACAGCTACTGGTTCGACTTTAACTCCCAGTTCAACATCGATGCTTCCAAGCGCGTCGGT
TACAGGAACATGATCGGAGACATTCCCGCCATGATTAACCCCGTGACGACCGGCAACCCCCTCGGCACCGGCGAATTCTTCAATCTTCCCATC
CCCCTCTTCTATTCCGAGGACTCGGGTCTGGCTCTTGCCGTCTCTGCCCTTCCTTTCAACGACATCAAGATAAACTACTGTCTCCGCAGGTGG
CAGGATCTTCTCGTCCTGAACGTGGGTGTTGGCGCCAACCCTCCCACTTTCGACGATGTTGTTCAGGTGAGCTACGACGCCACCTTCACCCTC
ATTTACAGCTCGAACGCTCCCGCCATCACCAATGTCGAGACTTGGTGCCACTACGCCGTTGTGCACAATGACGAACGTGTCAAGATGGGTAAG
AATCCCCGTGATATGGTCATCAAGCAGGTTCAAAAGGTCAACGAGACGACCATCAATCTTTCGCAGCTCAACGCTCTCATCCCGATTGATATC
CGTATCTCTCACGCCGTCGTTGGCTATTTCTACGCCATCAGGAACAGCTCCACTCCCGGCGAATGGTCCAACTACACCACCGAGCCCGCCTAT
GCCGGTCTTGACCCGCTTGAGGCTGCCCAGCTTGTGTACGAGTCGACTGCCCGTGTCAGCAATGGCTCTGATTATTACAGCCTTGTTGTGCCG
TGGTACTGGCACAAGTCGATCCCCGAGGAGACCGGCTACCACGCGTACTCGTACTCTCTGGAGACTTTCGCCTCTGATCCCAAGGGTTCGACC
AATTATTCCAAGCTCACCAACGTGTCGAACCAGTACGTTCCTTCGACCGCTGCCGTCAACGCCTCTGCTGGTGTGACCAACACCGGTATTCCG
ATTCCCTCAGCCACCAACCCTGCTGTGACTCAGCAGAACCAGACTTTCCAGCATATCTTCCGAGTCCTTAATTTCAACGTGTTGCGCCTCAGT
GGCGGCAGCTTGGGTCTTCCCATATTGTGA
>Kashiwazakivirus_2
ATGACTTCTGTTGTGTGTGGTACCAGAGTAACTGCTGCTTCCGGTTTCGTAGATTTGGCGACCTTCTCCGACTTGGAGGCGTACCTTTATGGT
GGTTGCTCGGCGGTCACCTATTTCGTGCGTGCCATCAAAAAGGCTAATTGGTTCTCTTTCCTCCCCGTTGTTCTCCGAAACATTTCGGGTCTT
CCCGGTTTCGGTAACGAATTCTCCGCGTCCGTGAATCGTTCCGGCGATTACGTCCTCAACACCTGGCTTCGCGTCCGTCTTCCTCTCATTGCT
ATCCGTCCGACCAATGCTGGTGGTGCCATCAACGCTAATGCCACCATCCGCTGGACCCGAAACTTCATGCACAATCTCGTTGAAAAGATCAAC
ATCACCTTCAATGATCTGATTGTCCACGAGTTTGACAGCTACTGGTTCGACTTTAACTCCCAGTTCAACATCGATGCTTCCAAGCGCGTCGGT
TACAGGAACATGATCGGAGACATTCCCGCCATGATTAACCCCGTGACGACCGGCAACCCCCTCGGCACCGGCGAATTCTTCAATCTTCCCATC
CCCCTCTTCTATTCCGAGGACTCGGGTCTGGCTCTTGCCGTCTCTGCCCTTCCTTTCAACGACATCAAGATCAACTACTGTCTCCGCAGGTGG
CAGGATCTTCTCGTCCTGAACGTGGGTGTTGGCGCCAACCCTCCCACTTTCGACGATGTTGTTCAGGTGAGCTACGACGCCACCTTCACCCTC
ATTTACAGCTCGAACGCTCCCGCCATCACCAATGTCGAGACTTGGTGCCACTACGCCGTTGTGCACAATGACGAGCGTGTCAAGATGGGTAAG
AATCCTCGTGATATGGTCATCAAGCAGGTTCAAAAGGTCAATGAGACGACCATCAATCTTTCGCAGCTCAACGCTCTCATCCCGATTGATATC
CGTATCTCTCACGCCGTCGTTGGCTATTTCTACGCCATCAGGAACAGCTCCACTCCCGGCGAATGGTCCAACTACACCACCGAGCCCGCCTAT
GCCGGTCTTGACCCGCTTGAGGCTGCCCAGCTTGTTTACGAGTCGACTGCCCGTGTCAGCAATGGCTCTGATTATTACAGCCTTGTTGTGCCG
TGGTACTGGCACAAGTCGATCCCCGAGGAGACCGGCTACCACGCGTACTCGTACTCTCTGGAGACTTTCGCCTCTGATCCCAAGGGTTCGACC
AATTATTCCAAGCTCACCAACGTGTCGAACCAGTACGTTCCTTCGACCGCTGCCGTCAACGCCTCTGCTGGTGTGACCAACACCGGTATTCCG
ATTCCCTCAGCCACCAACCCTGCTGTGACTCAGCAGAACCAGACTTTCCAGCATATCTTCCGAGTCCTTAATTTCAACGTGTTGCGCCTCAGT
GGCGGCAGCTTGGGTCTTCCCATATTGTGA
>Kashiwazakivirus_4
ATGACTTCTGTTGTGTGTGGTACCAGAGTAACTGCTGCTTCCGGTTTCGTAGATTTGGCGACCTTCTCCGACTTGGAGGCGTACCTTTATGGT
GGTTGCTCGGCGGTCACCTATTTCGTGCGTGCCATCAAAAAGGCTAATTGGTTCTCTTTCCTCCCCGTTGTTCTCCGAAACATTTCGGGTCTT
CCCGGTTTCGGTAACGAATTCTCCGCGTCCGTGAATCGTTCCGGCGATTACGTCCTCAACACCTGGCTTCGCGTCCGTCTTCCTCTCATTGCT
ATCCGTCCGACCAATGCTGGTGGTGCCATCAACGCTAACGCCACCATCCGCTGGACCCGAAACTTCATGCACAATCTTGTTGAAAAGATCAAC

ATCACCTTCAACGATCTGATTGTCCACGAGTTTGACAGCTACTGGTTCGACTTTAACTCCCAGTTCAACATCGATGCTTCCAAGCGCGTCGGT
TACAGGAACATGATCGGAGACATTCCCGCCATGATTAACCCCGTGACGACCGGCAACCCCCTCGGTACCGGCGAATTCTTCAATCTTCCCATC
CCCCTCTTCTATTCCGAGGACTCGGGTCTGGCTCTTGCCGTCTCTGCCCTTCCTTTCAACGACATCAAGATCAACTACTGTCTCCGCAGGTGG
CAGGATCTTCTCGTCCTGAACGTGGGTGTTGGCGCCAACCCTCCCACTTTCGACGATGTTGTTCAGGTGAGCTACGATGCCACCTTCACCCTC
ATTTACAGCTCGAACGCTCCCGCCATCACCAATGTCGAGACTTGGTGCCACTACGCCGTTGTGCACAATGACGAGCGTGTCAAGATGGGTAAG
AATCCCCGCGATATGGTCATCAAGCAGGTTCAAAAGGTCAACGAGACGACCATCAATCTTTCGCAGCTCAACGCTCTCATCCCGATTGATATC
CGTATCTCTCACGCCGTCGTTGGATACTTCTACGCCATCCGAAACAGCTCCACTCCCGGCGAATGGTCCAACTACACCACCGAGCCCGCCTAC
GCCGGTCTTGACCCGCTTGAGGCTGCCCAGCTTGTGTACGAGTCGACTGCCCGTGTCAGCAATGGCTCTGATTATTACAGCCTTGTTGTGCCG
TGGTACTGGCACAAGTCGATCCCCGAGGAGACCGGCTACCACGCGTACTCGTACTCGCTGGAGACTTTCGCCTCTGACCCCAAGGGTTCGACC
AATTATTCCAAGCTCACCAACGTGTCGAACCAGTACGTTCCTTCGACCGCTGCCGTCAACGCCTCTGCTGGTGTGACCAACACCGGTATTCCG
ATTCCCTCGGCCACCAACCCTGCTGTGCTTCAGCAGAACCAGACTTTCCAGCATATCTTCCGAGTCCTTAATTTCAACGTGTTGCGCCTCAGT
GGTGGCAGCTTGGGTCTTCCCATATTGTGA
>Kashiwazakivirus_5
ATGACTTCTGTTGTGTGTGGTACCAGAGTAACTGCTGCTTCCGGTTTCGTAGATTTGGCGACCTTCTCCGACTTGGAGGCGTACCTTTATGGT
GGTTGCTCGGCGGTCACCTATTTCGTGCGTGCCATCAAAAAGGCTAATTGGTTCTCTTTCCTCCCCGTTGTTCTCCGAAACATTTCGGGTCTT
CCCGGTTTCGGTAACGAATTCTCCGCGTCCGTGAATCGTTCCGGCGATTACGTCCTCAACACCTGGCTTCGCGTCCGTCTTCCTCTCATTGCT
ATCCGTCCGACCAATGCTGGTGGTGCCATCAACGCTAATGCCACCATCCGCTGGACCCGAAACTTCATGCACAATCTTGTTGAAAAGATCAAC
ATCACCTTCAATGATCTGATTGTCCACGAGTTTGACAGCTACTGGTTCGACTTTAACTCCCAGTTCAACATCGATGCTTCCAAACGCGTCGGT
TACAGGAACATGATCGGAGACATTCCCGCCATGATCAACCCCGTGACGACCGGCAACCCCCTCGGCACCGGCGAATTCTTCAATCTTCCCATC
CCCCTCTTCTATTCCGAGGACTCGGGTCTGGCTCTTGCCGTCTCTGCCCTTCCTTTCAACGACATCAAGATCAACTACTGTCTCCGCAGGTGG
CAGGATCTTCTCGTCCTGAACGTGGGCGTTGGCGCCAACCCTCCCACTCTCGACGATGTTGTTCAGGTGAGCTACGACGCCACTTTCACCCTT
ATTTACAGCTCGAACGCTCCCGCCATCACCAATGTCGAGACTTGGTGCCACTATGCCGTTGTGCACAATGACGAACGTGTCAAGATGGGTAAG
AATCCCCGTGATATGGTCATCAAGCAGGTTCAAAAGGTCAATGAGACGACCATCAACCTTTCGCAGCTCAACGCTCTCATCCCGATTGATATC
CGTATCTCTCACGCCGTCGTTGGATACTTCTACGCCATCCGAAACAGCTCCACTCCCGGCGAATGGTCCAACTACACCACCGAGCCCGCCTAC
GCCGGTCTTGACCCGCTTGAGGCTGCCCAGCTTGTGTATGAGTCGACCGCCCGTGTCAGCAACGGCTCTGATTATTACAGCCTTGTTGTGCCG
TGGTACTGGCACAAGTCGATCCCCGAGGAGACCGGCTACCACGCGTACTCGTACTCTCTGGAGACTTTCGCCTCTGATCCCAAGGGTTCGACC
AATTATTCCAAGCTCACCAATGTGTCGAACCAGTACGTTCCTTCGACCGCTGCCGTCAACGCCTCTGCTGGTGTGACCAACACCGGTATTCCG
ATTCCCTCAGCCACCAATCCTGCTGTGACTCAGCAGAACCAGACTTTCCAGCACATCTTCCGAGTCCTTAATTTCAACGTGTTGCGCCTCAGT
GGTGGCAGCTTGGGTCTTCCCATATTGTAG
>Kyotovirus_1
ATGACTTCTGTTATCTGTGGCACCCGCGTAACTGCTGCTTCCGGTTTTGTGGACTTGGCGACTTTCTCGGATTTGGAGGCTTACCTCTATGGT
GGTTGTTCCGCCGTCACCTATTTTGTGCGTGCCATCAAAAAGGCCAATTGGTTCTCCTTCCTCCCTGTTGTTCTTCGTAACATCTCGGGTCTT
CCCGGCTTCGGTTCAGAGTTCTCTGCTTCTGTGAATCGTTCCGGAGATTACGTCCTCAACACCTGGCTGCGTGTGCGTCTTCCTCTCGTGGCC
ATTCGTCCCACCAACACTGGTGGCGCCATCAACGCTAACGCCACCATTCGCTGGACCAGAAACTTCATGCACAATCTTGTGGAGAAGGTTAAC
ATCACTTTCAATGACCTCATCGTCCATGAGTTTGACAGCTACTGGTTCGACTTCAACTCGCAGTTCAACATCGACGCTTCCAAGCGCGTCGGT
TACAGGAACATGATCGGAGATATTCCGGCCATGATTAACCCTGTGACGACCGGCAACCCTCTCGGCACTGGCGAGTTCTTCAATCTTCCCATT
CCTCTCTTCTACACCGAGGATTCCGGTCTCGCCCTTGCTGTGTCAGCTCTTCCGTTCAACGACATCAAGATTAACTACTGTCTTCGCAGGTGG
CAGGATCTGATTGTCCTCAACGTGGGCGTCGGCGGTAACCCTCCCACTTATGACGACATTGTCCAGGTTTCTTACGACTCTACTTTCACCCTC
ATCTACAGTTCGAACGCTCCCGCCATCACCAATGTCGAGACCTGGTGTCACTACGCCGTCGTCCACAACGACGAACGCGTCAAGATGGGTAAG
AATCCCCGTGACATGGTCATCAAGCAGGTGCAAAAGGTCAACGAGACGACCATCAACCTTTCACAGCTCAACGCCCTTGTCCCCATCGACATT
CGCGTGTCTCATGCCGTCGTTGGATATTTCTACGCTATTCGAAACAGTTCGACCACTGGTGAATGGTCCAATTACACCACTGAACCAGCCTAT
GCCGGTCTTGACCCGCTCGAGGCTGCTCAGCTCGTGTACGAGTCGACTGCCCGTGTCAGCAACGGTTCTGACTATTACAGCCTGATGGTGCCG
TGGTATTGGCACAAGTCGATCCCCGAGGAGACGGGTTACCACGCATACTCTTATTCTCTCGACACCTTTGCTTCCGACCCCAAGGGTTCTACC
AATTATTCCAAACTCACCAACGTCTCGAACCAGTACGTTCCTTCGACCGCTGCTGTCAACGCTTCTGCTGGTGTGACCAACACCGGCATTCCG
ATTCCCTCGGCCACCAACCCCGCTGTGACTCAGCAGAACCAGACTTTCCAGCACATCTTCCGAGTTTTGAACTTTAACGTATTGCGCCTCAGT
GGCGGCTCCCTCGGATTGCCCGTCCTATGA
>Kyotovirus_2_5
ATGACTTCTGTTATCTGTGGCACCCGCGTAACTGCTGCTTCCGGTTTTGTGGACTTGGCGACTTTCTCGGATTTGGAGGCTTACCTCTATGGT

GGCTGTTCCGCCGTCACCTATTTTGTGCGTGCCATCAAAAAGGCCAATTGGTTCTCCTTCCTCCCTGTTGTTCTTCGTAACATCTCGGGTCTT
CCCGGCTTCGGTTCAGAGTTCTCTGCTTCTGTGAATCGTTCCGGAGATTACGTCCTCAACACCTGGCTGCGTGTGCGTCTTCCTCTCGTGGCC
ATTCGTCCCACCAACACTGGTGGCGCCATCAACGCTAACGCCACCATTCGCTGGACCAGAAACTTCATGCACAATCTTGTGGAGAAGGTTAAC
ATCACTTTCAATGACCTCATCGTCCATGAGTTTGACAGCTACTGGTTCGACTTCAACTCGCAGTTCAACATCGACGCTTCCAAGCGCGTCGGT
TACAGGAACATGATCGGAGATATTCCGGCCATGATTAACCCTGTGACGACCGGCAACCCTCTCGGCACTGGCGAGTTCTTCAATCTTCCCATT
CCTCTCTTCTACACCGAGGATTCCGGTCTCGCCCTTGCTGTGTCAGCTCTTCCGTTCAACGACATCAAGATTAACTACTGTCTTCGCAGGTGG
CAGGATCTGATTGTCCTCAACGTGGGCGTCGGCGGTAACCCTCCCACTTATGACGACATTGTCCAGGTTTCTTACGACTCGACTTTCACCCTC
ATCTACAGTTCGAACGCTCCCGCCATCACCAATGTCGAGACCTGGTGTCACTACGCCGTCGTCCACAACGACGAACGCGTCAAGATGGGTAAG
AATCCCCGTGACATGGTCATCAAGCAGGTGCAAAAGGTCAACGAGACGACCATCAACCTTTCACAGCTCAACGCCCTTGTCCCCATCGACATT
CGCGTGTCTCATGCCGTCGTTGGATATTTCTACGCTATTCGAAACAGTTCGACCACTGGTGAATGGTCCAATTACACCACTGAACCAGCCTAT
GCCGGTCTTGACCCGCTCGAGGCTGCTCAGCTCGTGTACGAGTCGACTGCCCGTGTCAGCAACGGTTCTGACTATTACAGCCTGATGGTGCCG
TGGTATTGGCACAAGTCGATCCCCGAGGAGACGGGTTACCACGCATACTCTTATTCTCTCGACACCTTTGCTTCTGACCCCAAGGGTTCGACC
AATTATTCCAAGCTCACTAACGTGTCGAACCAGTACGTTCCTTCGACCGCTGCTGTCAACGCTTCTGCTGGTGTGACCAACACCGGCATTCCG
ATTCCCTCGGCCACCAACCCCGCTGTGACTCAGCAGAACCAGACTTTCCAGCACATCTTCCGAGTTTTGAACTTTAACGTATTGCGCCTCAGT
GGCGGCTCCCTCGGATTGCCCGTCCTATGA
>Kyotovirus_3
ATGACTTCTGTTATCTGTGGCACCCGCGTAACTGCTGCTTCCGGTTTTGTGGACTTGGCGACTTTCTCGGATTTGGAGGCTTACCTCTATGGT
GGTTGTTCCGCCGTCACCTATTTTGTGCGTGCCATCAAAAAGGCCAATTGGTTCTCCTTCCTCCCTGTTGTTCTTCGTAACATCTCGGGTCTT
CCCGGCTTCGGTTCAGAGTTCTCTGCTTCTGTGAATCGTTCCGGAGATTACGTCCTCAACACCTGGCTGCGTGTGCGTCTTCCTCTCGTGGCC
ATTCGTCCCACCAACACTGGTGGCGCCATCAACGCTAACGCCACCATTCGCTGGACCAGAAACTTCATGCACAATCTTGTGGAGAAGGTTAAC
ATCACTTTTAATGACCTCATCGTCCATGAGTTTGACAGCTACTGGTTCGACTTCAACTCGCAGTTCAACATCGACGCTTCCAAGCGCGTCGGT
TACAGGAACATGATCGGAGATATTCCGGCCATGATTAACCCTGTGACGACCGGCAACCCTCTCGGCACTGGCGAGTTCTTCAATCTTCCCATT
CCTCTCTTCTACACCGAGGATTCCGGTCTCGCCCTTGCTGTGTCAGCTCTTCCGTTCAACGACATCAAGATTAACTACTGTCTTCGCAGGTGG
CAGGATCTGATTGTCCTCAACGTGGGCGTCGGCGGTAACCCTCCCACTTATGACGACATTGTCCAGGTTTCTTACGACTCTACTTTCACCCTC
ATCTACAGTTCGAACGCTCCCGCCATCACCAATGTCGAGACCTGGTGTCACTACGCCGTCGTCCACAACGACGAACGCGTCAAGATGGGTAAG
AATCCCCGTGACATGGTCATCAAGCAGGTGCAAAAGGTCAACGAGACGACCATCAACCTTTCACAGCTCAACGCCCTTGTCCCCATCGACATT
CGCGTGTCTCATGCCGTCGTTGGATATTTCTACGCTATTCGAAACAGTTCGACCACTGGTGAATGGTCCAATTACACCACTGAACCAGCCTAT
GCCGGTCTTGACCCTCTTGAGGCCGCTCAGCTCGTGTACGAGTCGACTGCCCGTGTCAGCAACGGTTCTGACTATTACAGCCTGATGGTGCCG
TGGTATTGGCACAAGTCGATCCCCGAGGAGACGGGTTACCACGCATACTCTTATTCTCTCGACACCTTTGCTTCCGACCCCAAGGGTTCTACC
AATTATTCCAAACTCACCAACGTCTCGAACCAGTACGTTCCTTCGACCGCTGCTGTCAACGCTTCTGCTGGTGTGACCAACACCGGCATTCCG
ATTCCCTCGGCCACCAACCCCGCTGTAACTCAGCAGAACCAGACTTTCCAGCACATCTTCCGAGTTTTGAACTTTAACGTATTGCGCCTCAGT
GGCGGCTCCCTCGGATTGCCCGTCCTATGA
>Kyotovirus_4_6
ATGACTTCTGTTATCTGTGGCACCCGCGTAACTGCTGCTTCCGGTTTTGTGGACTTGGCGACTTTCTCGGATTTGGAGGCTTACCTCTATGGT
GGTTGTTCCGCCGTCACCTATTTTGTGCGTGCCATCAAAAAGGCCAATTGGTTCTCCTTCCTCCCTGTTGTTCTTCGTAACATCTCGGGTCTT
CCCGGCTTCGGTTCAGAGTTCTCTGCTTCTGTGAATCGTTCCGGAGATTACGTCCTCAACACCTGGCTGCGTGTGCGTCTTCCTCTCGTGGCC
ATTCGTCCCACCAACACTGGTGGCGCCATCAACGCTAACGCCACCATTCGCTGGACCAGAAACTTCATGCACAATCTTGTGGAGAAGGTTAAC
ATCACTTTCAATGACCTCATCGTCCATGAGTTTGACAGCTACTGGTTCGACTTCAACTCGCAGTTCAACATCGACGCTTCCAAGCGCGTCGGT
TACAGGAACATGATCGGAGATATTCCGGCCATGATTAACCCTGTGACGACCGGCAACCCTCTCGGCACTGGCGAGTTCTTCAATCTTCCCATT
CCTCTCTTCTACACCGAGGATTCCGGTCTCGCCCTTGCTGTGTCAGCTCTTCCGTTCAACGACATCAAGATTAACTACTGTCTTCGCAGGTGG
CAGGATCTGATTGTCCTCAACGTGGGCGTCGGCGGTAACCCTCCCACTTATGACGACATTGTCCAGGTTTCTTACGACTCTACTTTCACCCTC
ATCTACAGTTCGAACGCTCCCGCCATCACCAATGTCGAGACCTGGTGTCACTACGCCGTCGTCCACAACGACGAACGCGTCAAGATGGGTAAG
AATCCCCGTGACATGGTCATCAAGCAGGTGCAAAAGGTCAACGAGACGACCATCAACCTTTCACAGCTCAACGCCCTTGTCCCCATCGACATT
CGCGTGTCTCATGCCGTCGTTGGATACTTCTACGCTATCCGAAACAGTTCGACCACTGGTGAATGGTCCAATTACACCACTGAACCAGCCTAT
GCCGGTCTTGACCCGCTCGAGGCCGCTCAGCTCGTGTACGAGTCGACTGCCCGTGTCAGCAACGGTTCTGACTATTACAGCCTGATGGTGCCG
TGGTATTGGCACAAGTCGATCCCCGAGGAGACGGGTTACCACGCGTACTCTTATTCTCTCGACACCTTTGCTTCCGACCCCAAGGGTTCGACC
AATTATTCCAAGCTCACCAACGTCTCGAACCAATACGTTCCTTCGACCGCTGCTGTCAACGCTTCTGCTGGCGTGACCAACACCGGCATTCCG
ATTCCCTCGGCCACGAACCCTGCCGTGACTCAGCAGAACCAGACTTTCCAGCACATCTTCCGAGTTTTGAACTTTAACGTGCTTCGTTTGTCT

GGAGGAAGCTTGGGTCTTCCAGTATTGTAG
>Kyotovirus_7
ATGACTTCTGTTATCTGTGGCACCCGCGTAACTGCTGCTTCCGGTTTTGTGGACTTGGCGACTTTCTCGGATTTGGAGGCTTACCTCTATGGT
GGCTGTTCCGCCGTCACCTATTTTGTGCGTGCCATCAAAAAGGCCAATTGGTTCTCCTTCCTCCCTGTTGTTCTCCGTAACATCTCGGGTCTT
CCCGGCTTCGGTTCAGAGTTCTCTGCTTCTGTGAATCGTTCCGGAGATTACGTCCTCAACACCTGGCTGCGTGTGCGTCTTCCTCTCGTGGCC
ATTCGTCCCACCAACACTGGTGGCGCCATCAACGCTAACGCCACCATTCGCTGGACCAGAAACTTCATGCACAATCTTGTGGAGAAGGTTAAC
ATCACTTTCAATGACCTCATCGTCCATGAGTTTGACAGCTACTGGTTCGACTTCAACTCGCAGTTCAACATCGACGCTTCCAAGCGCGTCGGT
TACAGGAACATGATCGGAGATATTCCGGCCATGATTAACCCTGTGACGACCGGCAACCCTCTCGGCACTGGCGAGTTCTTCAATCTTCCCATT
CCTCTCTTCTACACCGAGGATTCCGGTCTCGCCCTTGCTGTGTCAGCTCTTCCGTTCAACGACATCAAGATTAACTACTGTCTTCGCAGGTGG
CAGGATCTGATTGTCCTCAACGTGGGCGTCGGCGGTAACCCTCCCACTTATGACGACATTGTTCAGGTTTCTTACGACTCGACTTTCACCCTC
ATCTACAGTTCGAACGCTCCCGCCATCACCAATGTCGAGACCTGGTGTCACTACGCCGTCGTCCACAACGACGAACGTGTCAAGATGGGTAAG
AATCCCCGTGACATGGTCATCAAGCAGGTGCAAAAGGTCAACGAGACGACCATCAACCTTTCACAGCTCAACGCCCTTGTCCCCATCGACATT
CGCGTGTCTCATGCCGTTGTTGGATACTTCTACGCTATCCGAAACAGTTCGACCACTGGTGAATGGTCCAATTACACCACTGAACCAGCCTAT
GCCGGTCTTGACCCGCTCGAGGCTGCTCAGCTCGTGTACGAGTCGACTGCCCGTGTCAGCAACGGTTCTGACTATTACAGCCTGATGGTGCCG
TGGTATTGGCACAAGTCGATCCCCGAGGAGACGGGTTACCACGCATACTCTTATTCTCTCGACACCTTTGCTTCTGACCCCAAGGGTTCGACC
AATTATTCCAAGCTCACTAACGTGTCGAACCAGTACGTTCCTTCGACCGCTGCTGTCAACGCTTCTGCTGGTGTGACCAACACCGGCATTCCG
ATTCCCTCGGCCACCAACCCCGCTGTGACTCAGCAGAACCAGACTTTCCAGCACATCTTCCGAGTTTTGAACTTTAACGTGCTTCGTTTGTCT
GGAGGAAGCTTGGGTCTTCCAGTATTGTAG

Supplementary Data 2. Partial sequences of D5-like helicase-primase genes of newly isolated 15 viruses.

>Hokutovirus_1_2
ACGATGAAAAGATGCCTTGATATGCAAGCGAAACTTCAAATGGATAGTTTTGCCACAGGGGTCATGAGAATGTGCAAAAGACTTTTCCTGAAC
GAGAAGTTTCTCGAGAAGCTTGATGAAAACAGAGACATCATCGGAATGGAGGATGGCGTCGTCGAC
>Kashiwazakivirus_1_2
ACGATGAAAAGGTGCCTTGATATGCAGGCGAAACTTCAGATGGATAGTTTTGCCACGGGAGTCATGAGAATGTGCAAGAGGCTTTTCCTGAAC
GAGAAGTTTCTCGAAAAGTTAGACGAGAACAGAGACATCATCGGAATGGAGGATGGCGTCGTTGAC
>Kashiwazakivirus_3_6
ACGATGAAAAGGTGCCTTGATATGCAAGCGAAACTTCAGATGGATAGTTTTGCCACGGGAGTCATGAGAATGTGCAAGAGGCTTTTCCTGAAT
GAAAAGTTTTTCGAAAAGCTAGACGAGAACAGAGACATCATCGGGATGGAGGATGGCGTCGTTGAC
>Kashiwazakivirus_4_5
ACGATGAAAAGGTGCCTTGATATGCAGGCGAAACTTCAGATGGATAGTTTTGCCACGGGAGTCATGAGAATGTGCAAGAGGCTTTTCCTGAAT
GAGAAGTTTTTCGAAAAGCTAGACGAGAACAGAGACATCATCGGGATGGAGGATGGCGTCGTTGAC
>Kyotovirus_1_2_4_5_6_7
ACCATCAAGAAATGTGTGGACATCCAATCCAAGCTCCAGATGGATGGATTTTCTCAGGGAGTGATGAAGATGTGCAAGAGACTGTTTTTGAAC
GAGCATTTCCTCTCCAAACTCGACGAAAACAGAGACCTCTTGGGGATGGAGGACGGAGTTTGTGAC
>Kyotovirus_3
ACCATCAAGAAATGTGTGGACATCCAATCCAAACTTCAGATGGATGGATTTTCTCAGGGAGTGATGAAGATGTGCAAGAGACTGTTTTTGAAT
GAGCATTTCCTCTCCAAACTCGACGAAAACAGAGACCTCTTGGGGATGGAGGACGGAGTTTGTGAC
